# Knowledge structure and thematic evolution of host response-oriented sepsis research: a multi-database bibliometric and LDA topic modeling study

**DOI:** 10.3389/fimmu.2026.1872111

**Published:** 2026-07-01

**Authors:** Congcong Qin, Weiwei Wang, Qinyuan Du, Li Kong, Guochen Li

**Affiliations:** 1 Institute of Chinese Medical Literature and Culture, Shandong University of Traditional Chinese Medicine, Jinan, Shandong, China; 2 The First Clinical Medical College, Shandong University of Traditional Chinese Medicine, Jinan, Shandong, China; 3 Affiliated Hospital of Shandong University of Traditional Chinese Medicine, Jinan, Shandong, China

**Keywords:** bibliometric analysis, host response, latent Dirichlet allocation, precision stratification, sepsis, topic evolution

## Abstract

**Objective:**

To characterize the knowledge structure, thematic domains, and temporal evolution of host response-oriented sepsis research and to summarize its major research themes, organizational patterns, and temporal shifts.

**Methods:**

Publications related to host response, immune phenotypes, endotypes, and multimarker stratification in adult sepsis were retrieved from Web of Science Core Collection, Scopus, and PubMed. A total of 5,839 records were identified. After exclusion of two records with missing titles, 5,837 records entered a two-stage deduplication workflow based on DOI matching followed by metadata-adjudicated normalized-title matching, yielding 2,974 unique publications. Titles, abstracts, author keywords, and index keywords were concatenated to construct the textual corpus. Bibliometric analysis and latent Dirichlet allocation topic modeling were used to identify thematic domains; each publication was assigned to its dominant topic based on the maximum document-topic posterior probability from the final eight-topic (K = 8) model. Topic-specific annual distributions from 2000 to 2025 were analyzed to characterize temporal evolution.

**Results:**

The annual publication output increased steadily after 2000 and entered a marked expansion phase after 2016. The literature involved broad international participation and was published across immunology, critical care, infectious disease, and translational medicine journals. Across K = 4 to 12 solutions evaluated by coherence, perplexity, seed stability, and minimum topic size, an eight-topic model offered the best balance, and eight thematic domains were identified. The largest domains were inflammatory and innate-immune signaling (n=768, 25.8%), clinical management and precision medicine (n=573, 19.3%), and organ dysfunction, endothelial injury, and coagulation (n=392, 13.2%). Temporal analysis based on annual publication counts showed that inflammatory signaling and clinical management remained foundational, whereas transcriptomics, diagnostic and prognostic biomarkers, and ICU outcome themes showed apparent recent increases at the macro-thematic level. Preprocessing sensitivity analyses indicated that macro-level themes were interpretable but preprocessing-dependent.

**Conclusion:**

Host response-oriented sepsis research has become organized around several distinct but interconnected thematic domains. Its publication activity shows a descriptive shift from traditional inflammation-centered investigation toward molecular characterization, precision stratification, and individualized management. Transcriptomics, biomarkers, prognostic stratification, and early diagnosis may remain important directions for future research.

## Introduction

Sepsis is a life-threatening syndrome characterized by organ dysfunction resulting from a dysregulated host response to infection (1, 2). Despite continuous advances in intensive care, infection control, and organ support, sepsis remains associated with substantial morbidity, mortality, and healthcare utilization (3, 4). The pathophysiology of sepsis involves complex interactions among invading pathogens, host immune responses, inflammatory cascades, coagulation disturbances, microcirculatory dysfunction, metabolic reprogramming, and multi-organ injury (5, 6). This intrinsic heterogeneity complicates early recognition, prognostic stratification, treatment-response prediction, and clinical trial design (7, 8).

Growing recognition of host-response heterogeneity has shifted the research focus beyond conventional infection control and broad anti-inflammatory strategies (9, 10). Increasing attention has been directed toward dysregulated host response, immune phenotypes, endotypes, molecular biomarkers, and multimarker-based stratification (11, 12). These concepts provide a mechanistic framework for explaining why patients with apparently similar infectious triggers may demonstrate divergent immune activation states, organ dysfunction patterns, and clinical outcomes (13, 14).

Although sepsis-related literature has expanded rapidly, the field remains fragmented. Existing studies often focus on isolated pathways, single biomarkers, or individual clinical questions, whereas prior bibliometric analyses have generally addressed sepsis research as a broad domain rather than host response-oriented sepsis as a distinct and increasingly precision-medicine-oriented subfield (15). However, the latent thematic structure, organizational characteristics, and temporal evolution of this subdomain have not been systematically mapped.

Accordingly, this study integrated bibliometric analysis with latent Dirichlet allocation topic modeling to characterize the knowledge structure and temporal evolution of host response-oriented sepsis research (16, 17). By constructing a cross-database, strictly deduplicated dataset, we aimed to identify the major thematic domains, quantify their relative sizes, describe organizational patterns, and examine temporal shifts from classical inflammatory mechanisms toward molecular characterization, precision stratification, and individualized decision-making.

## Materials and methods

### Data sources and search strategy

Bibliographic records were retrieved from Web of Science Core Collection, Scopus, and PubMed following established bibliometric practice (18). The search combined a sepsis axis (sepsis or septic shock) with a host-response axis (host response, dysregulated host response, host immune response, immune phenotype, immune endotype, endotype, and biomarker- or multimarker-based stratification, phenotyping, or endotyping). Neonatal, pediatric, and animal-related records were excluded at the retrieval stage to maintain a focus on adult clinical and translational host response-oriented sepsis research. Neonatal and pediatric sepsis differ in immune developmental background, diagnostic frameworks, pathogen distribution, and therapeutic context, whereas animal-only studies, although essential for mechanistic discovery, would shift the corpus toward model-specific experimental mechanisms rather than adult clinical and translational host-response research. The findings should therefore be interpreted as applying to adult host response-oriented sepsis literature. The final search date was 29 March 2026; complete database-specific Boolean search strings are provided in **Supplementary Table 1**.

### Data integration and strict deduplication

A total of 5,839 bibliographic records were retrieved across the three databases. Records were harmonized into a standardized dataset containing titles, abstracts, keywords, publication years, digital object identifiers, and source databases.

After removal of two records with missing titles, 5,837 records entered the two-stage deduplication workflow. First, duplicate entries were removed by DOI matching after normalization (lowercasing; removal of resolver and doi prefixes; trimming of trailing punctuation; empty DOIs were not used for matching), which removed 2,789 records and left 3,048. Second, residual duplicates were removed by metadata-adjudicated normalized-title matching: within each group of records sharing an identical normalized title, two records were treated as the same publication only if their DOIs were equivalent after format normalization (case, hyphen/underscore, and leading-zero differences) or they shared the same first author and publication year; otherwise they were retained as distinct works. This step removed 74 records and avoids both naive title merging (which collapses distinct generic-title works such as book chapters titled Sepsis or Septic shock) and double-counting of DOI format variants. The procedure yielded 2,974 unique publications for downstream analyses; per-group adjudication is provided in **Supplementary Table 8**.

### Corpus construction and preprocessing

For text mining, the title, abstract, author keywords, and index keywords/keywords-plus of each publication were concatenated to construct a unified textual corpus. All text was converted to lowercase, and URLs, non-alphabetic symbols, and redundant spaces were removed. Standard English stopwords and demographic MeSH/check-tag noise terms (for example, male, female, humans, adult, aged, child, infant, animals, mice, rats) were removed, and tokens shorter than three characters were discarded.

Lemmatization was not applied in the primary pipeline, to preserve biomedical terms and phrase-level specificity. Because the search strategy itself was centered on terms such as sepsis, septic, and shock, these query-defining terms were removed from the corpus to prevent them from dominating the model. After filtering terms occurring in fewer than 5 documents (no_below = 5) or in more than 50% of documents (no_above = 0.5), the corpus was represented as a bag-of-words document-term matrix with a vocabulary of 6,083 terms and dimensions of 2,974 x 6,083.

### Bibliometric analysis

Bibliometric analysis was performed to describe annual publication output, institutional productivity, author productivity, country collaboration patterns, journal distribution, and keyword structures (18, 19). Before ranking, journal, country, institution, and author metadata were standardized: journal titles were standardized by case normalization, abbreviation harmonization, and variant-to-canonical mapping (for example, Shock, SHOCK, and Shock (Augusta, Ga.) were consolidated into Shock); country names were harmonized (for example, USA and United States of America to United States); institution names were harmonized using representative variant-to-canonical mappings; and author names were standardized to a surname-initial format, with common surname-initial strings such as Wang Y and Zhang Y treated cautiously as potentially ambiguous aggregates. Web of Science continuation-line author parsing was corrected and verified. Country productivity used full counting of affiliation-derived country occurrences, with each country counted once per record; the country collaboration network was constructed from co-occurring countries within the same record, with edge thickness representing collaboration intensity (20, 21). Bibliometric indicators and science-mapping visualizations were designed according to established bibliometric principles and were conceptually consistent with commonly used tools such as bibliometrix, CiteSpace, and VOSviewer (19–21). However, all rankings, networks, and visualizations in the present study were generated using a custom Python pipeline based on networkx and matplotlib. Representative normalization mappings are provided in **Supplementary Table 4**.

Keyword co-occurrence and keyword overlay visualizations were used to characterize research hotspots and their temporal migration. Network visualizations were based on filtered high-frequency nodes to preserve readability while retaining the major structural relationships among terms.

### LDA topic modeling and topic assignment

Latent Dirichlet allocation was implemented in Python using the LdaModel class from gensim (version 4.4.0) (16, 17). To select the number of topics, models were fitted for K = 4 to 12, each repeated across three random seeds (42, 7, and 2026), with passes = 20, iterations = 200, alpha = auto, and eta = auto, and evaluated using c_v topic coherence, log-perplexity bound, top-20 term seed stability (mean pairwise Jaccard overlap across seeds after optimal topic matching), and minimum topic size. The highest coherence occurred at K = 12 (c_v = 0.5272) and the highest seed stability at K = 4 (0.4893); at K = 8, c_v = 0.5184, seed stability = 0.3775, and the minimum topic size was 83, with coherence at a local maximum and seed stability declining at K of 9 or more, where higher-K solutions showed increasingly fragmented topics. Here, the minimum topic size of 83 refers to the model-selection diagnostic averaged across seeds, whereas the final random_state = 42 model yielded a minimum dominant-topic count of 75. The eight-topic solution was therefore selected as a balanced compromise across coherence, stability, topic-size balance, and interpretability rather than as the optimum on any single metric. The full K = 4 to 12 diagnostics are summarized in **Supplementary Table 3**.

The final eight-topic model was trained with random_state = 42, passes = 20, iterations = 200, alpha = auto, and eta = auto. Preprocessing, model, and network-visualization parameters are summarized in **Supplementary Table 6**.

After model fitting, each publication was assigned to the topic with the highest document-topic posterior probability without manual reassignment. Topic labels were assigned by author-team consensus after reviewing high-weight model terms, high-probability representative publications, and semantic coherence; no quantitative inter-rater reliability statistic was calculated.

### Preprocessing sensitivity analyses

To assess robustness to preprocessing choices, the primary pipeline was compared with four alternatives at K = 8: (A) morphological normalization (stemming); (B) retaining shock while removing sepsis and septic; (C) retaining all query-defining terms; and (D) not removing demographic noise terms. Agreement with the primary pipeline was quantified by top-20 term overlap and dominant-topic agreement after optimal topic matching. The configurations and results are reported in **Supplementary Table 7**.

### Temporal evolution analysis

Annual topic-specific publication counts were calculated by linking dominant topic assignments with publication years. The annual topic displays were restricted to the 2000–2025 window, which contained 2,674 records. Records outside this display window were retained in the overall corpus and topic-size calculations but were not included in the annual trend displays. To make contemporary topic divergence easier to interpret, the principal trend figure focused on 2010–2025, whereas the full 2000–2025 range was retained in the **Supplementary Material**.

## Results

### Literature retrieval and dataset construction

**Figure 1:**
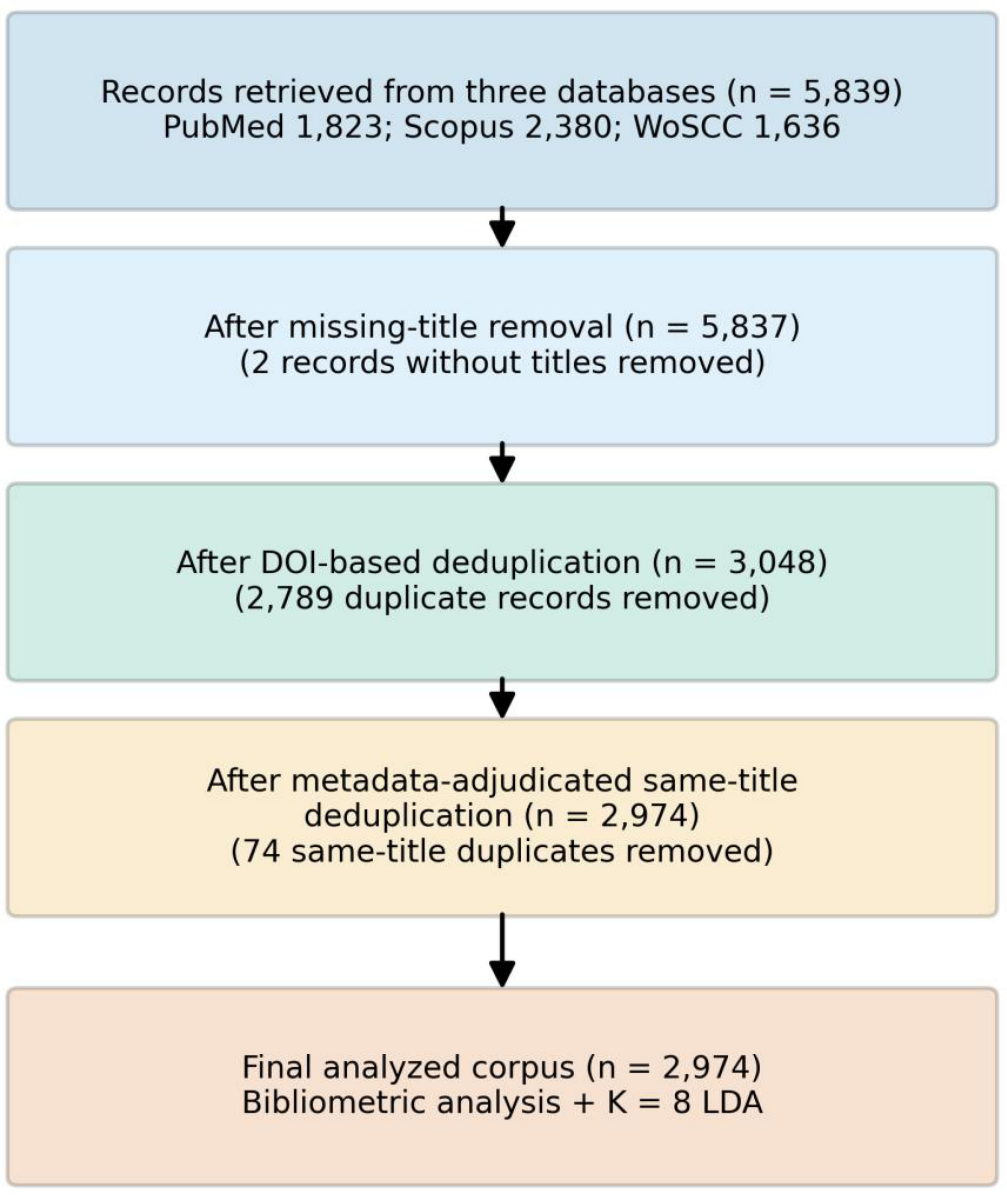
Workflow of literature retrieval, cleaning, strict deduplication, and final inclusion. The diagram summarizes the construction of the final analyzed corpus. A total of 5,839 records were retrieved from Web of Science Core Collection, Scopus, and PubMed. After exclusion of two records with missing titles, 5,837 records entered DOI matching (2,789 duplicates removed) followed by metadata-adjudicated normalized-title matching (74 duplicates removed), resulting in 2,974 unique publications for downstream bibliometric analysis and LDA topic modeling.

Records from the three databases were harmonized and deduplicated to reduce cross-database redundancy while retaining broad coverage of host response-oriented sepsis literature. As shown in **Figure 1**, 5,839 records were initially retrieved from Web of Science Core Collection, Scopus, and PubMed; after removal of two records with missing titles, 5,837 records entered DOI matching (2,789 duplicates removed) followed by metadata-adjudicated normalized-title matching (74 duplicates removed), leaving 2,974 unique publications for downstream analysis.

### General publication characteristics

**Figure 2:**
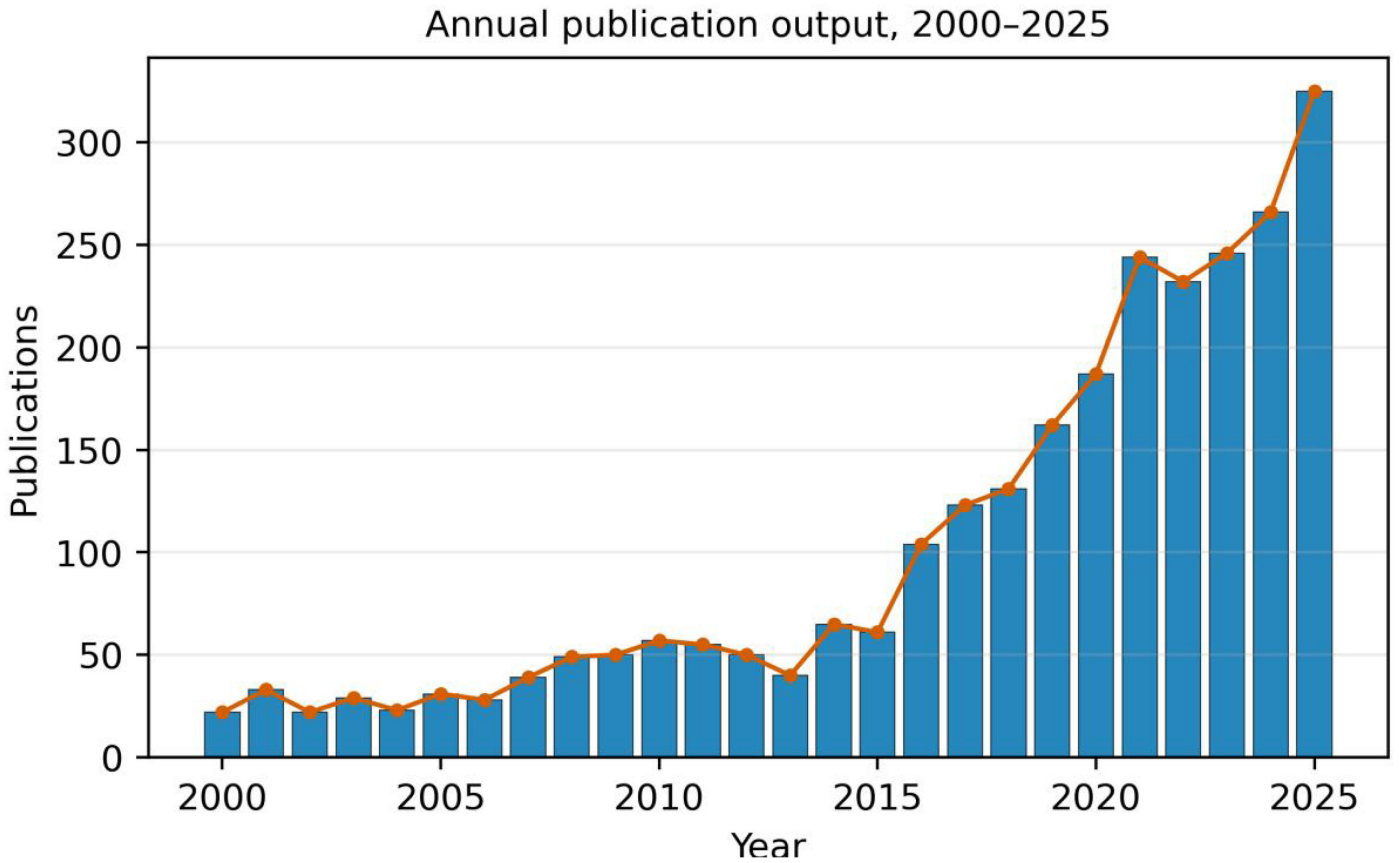
Annual publication trend of host response-oriented sepsis research, 2000–2025. Annual publication counts were calculated from the strictly deduplicated final dataset and displayed for 2000–2025. The trend shows a gradual increase after 2000 and a more evident expansion after 2016. Counts for recent years should be interpreted cautiously because of possible indexing delay and incomplete database coverage.

Annual publication counts suggested a relatively slow early growth phase, followed by a clear increase in research activity during the most recent decade. Publication output increased steadily after 2000 and showed a more pronounced expansion after 2016, suggesting sustained growth and increasing thematic differentiation in host response-oriented sepsis research (**Figure 2**).

### Institutional, author, geographical, and journal patterns

**Figure 3:**
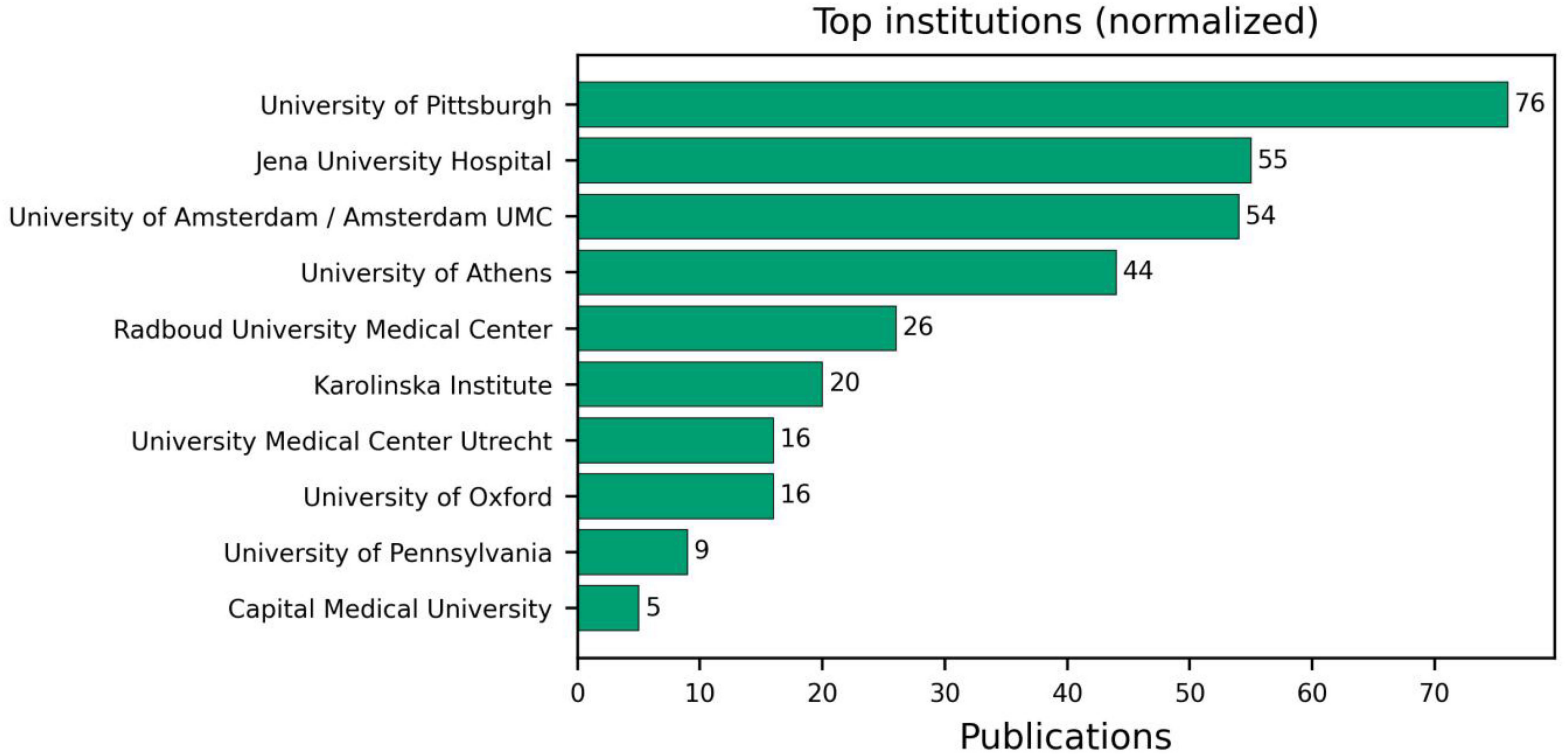
Top institutions by publication output in host response-oriented sepsis research. Institutional productivity was calculated from standardized affiliation information in the final dataset. The image ranks the leading institutions according to publication output, reflecting major organizational contributors to research on sepsis host response, immune heterogeneity, biomarkers, and precision stratification.

**Figure 4:**
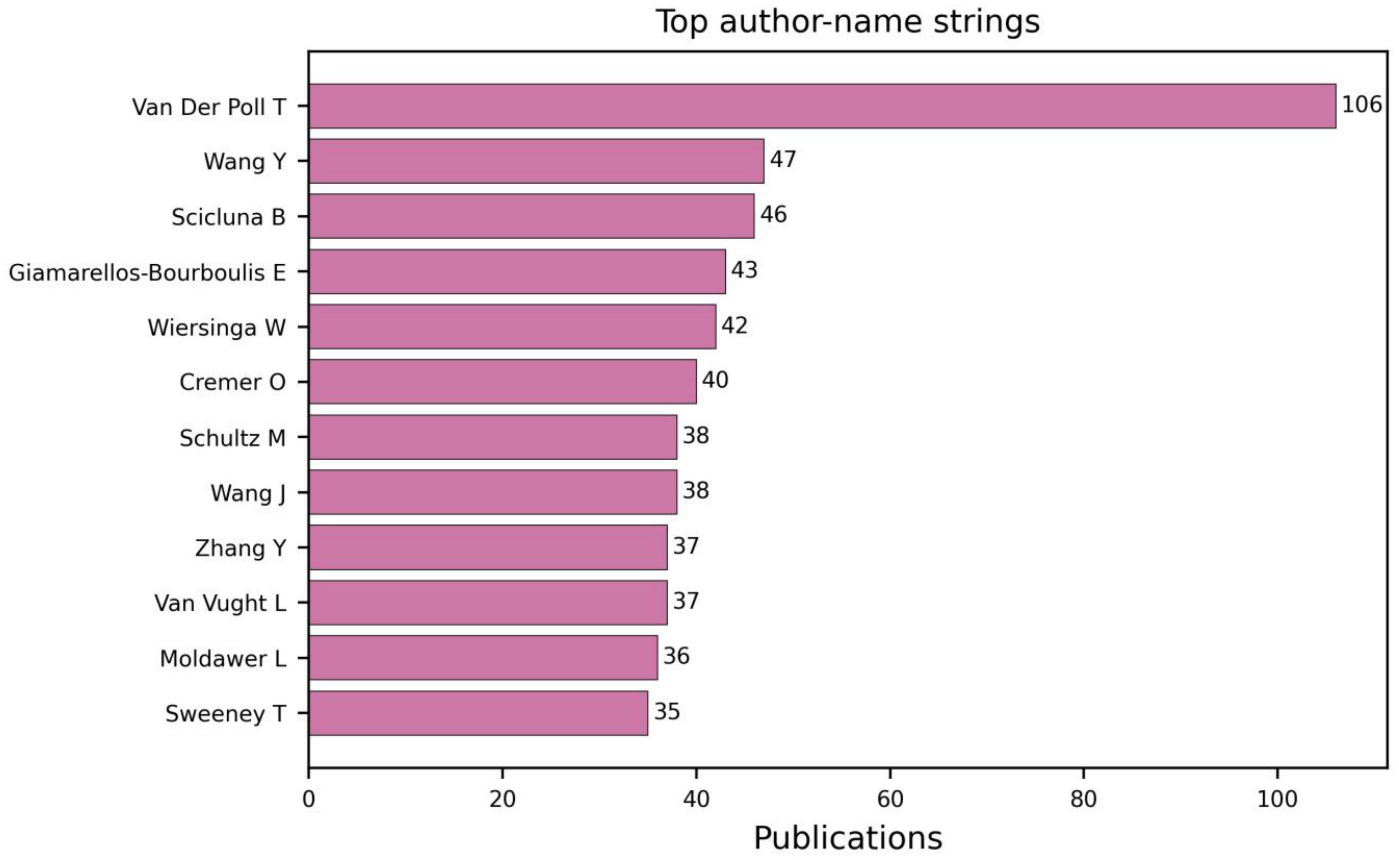
Top author-name strings by publication output in host response-oriented sepsis research. Author productivity was calculated from standardized author fields in the final dataset. The image shows the leading standardized author-name strings in the host response-oriented sepsis literature and highlights those with sustained output in sepsis immunology, molecular profiling, and stratification-related research.

Productivity analysis, performed after metadata normalization, identified recurrent research centers and investigators with sustained contributions to sepsis immunology, host-response profiling, and translational critical care research. The leading institutions included the University of Pittsburgh, Jena University Hospital, the University of Amsterdam/Amsterdam UMC, and the University of Athens (**Figure 3**), whereas the most productive author-name strings were van der Poll T (n=106), Wang Y (n=47), and Scicluna B (n=46) (**Figure 4**). Because the records relied on abbreviated author names, common surname-initial strings such as Wang Y and Zhang Y (n=37) represent potentially ambiguous aggregates rather than confirmed single individuals; key rankings were recalculated after normalization and after correcting Web of Science continuation-line author parsing.

**Figure 5:**
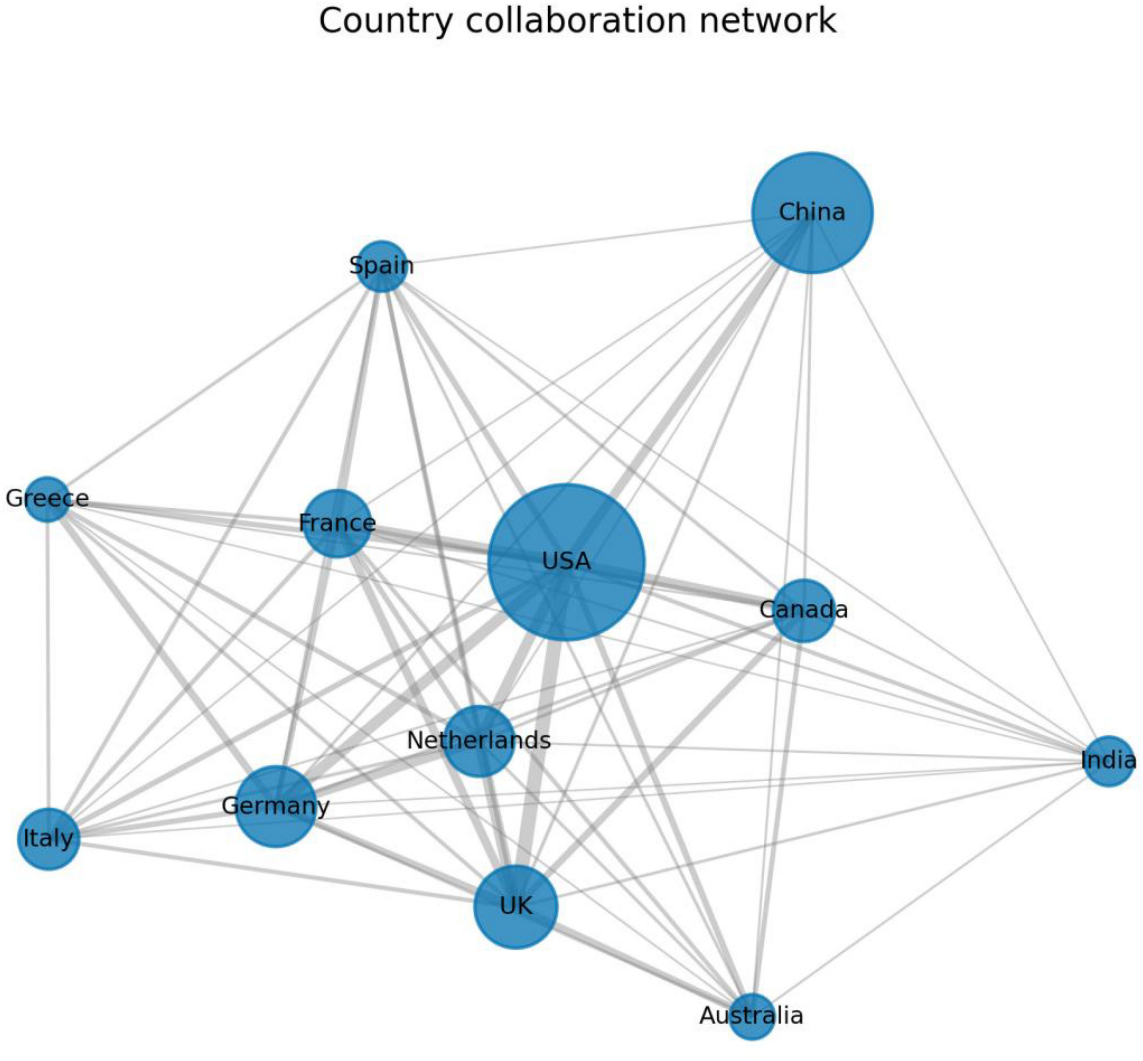
Country collaboration network in host response-oriented sepsis research. Countries were identified from affiliation information in the final dataset. Node size represents publication output, and edges indicate co-authorship-based collaboration between countries. Thicker edges denote stronger collaboration intensity, reflecting the international structure of research on host-response heterogeneity, biomarker validation, and sepsis stratification.

At the geographical level, the United States (n=939), China (n=557), the United Kingdom (n=262), and Germany (n=249) were the leading contributing countries. The country collaboration network showed several stable international collaboration clusters, consistent with the multicenter and cross-regional nature of biomarker validation, endotype discovery, and translational critical care studies (**Figure 5**). The publication output of leading countries is provided in **Supplementary Figure 2** to complement the collaboration network analysis.

**Figure 6:**
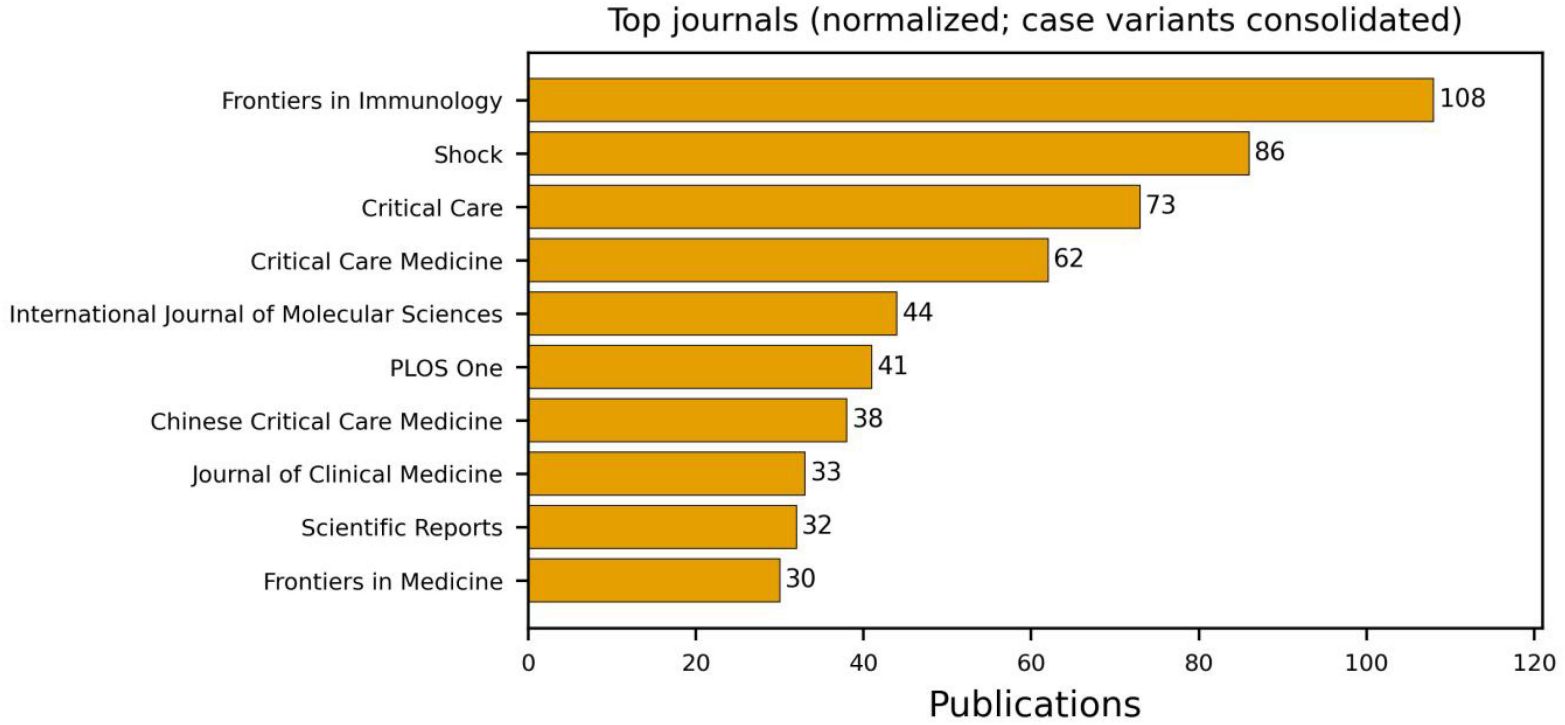
Top journals by publication output in host response-oriented sepsis research. Journals were ranked according to the number of publications in the strictly deduplicated dataset. The distribution shows that this field is mainly disseminated through journals related to immunology, critical care medicine, infectious diseases, and translational biomedical research.

Journal distribution indicated that the literature was published across immunology, critical care, infectious disease, and translational medicine journals. Frontiers in Immunology, Shock, and Critical Care were the leading journals by publication output, indicating the interdisciplinary positioning of this research field (**Figure 6**). To further link geographical contribution, journal dissemination, and thematic structure, the three-field relationship among countries, journals, and LDA-defined thematic domains is shown in **Supplementary Figure 3**. Extended bibliometric ranking tables are provided in **Supplementary Table 4**.

### Keyword structure and research hotspots

**Figure 7:**
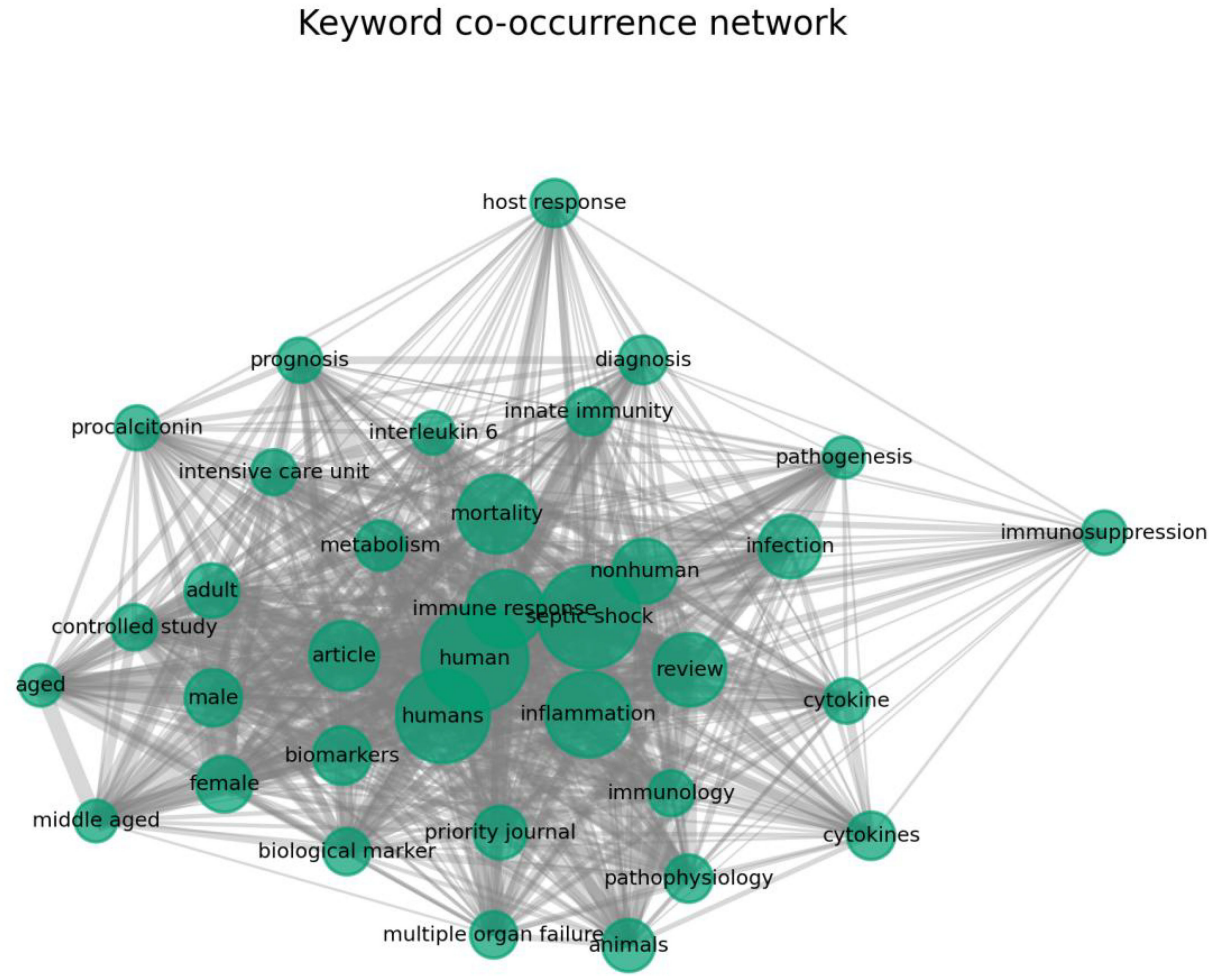
Keyword co-occurrence network of host response-oriented sepsis research. The network was constructed from high-frequency keywords in the final dataset. Node size represents keyword frequency, and edge strength represents co-occurrence intensity. The image shows the major keyword clusters and their structural relationships, including inflammation, immune regulation, biomarkers, organ dysfunction, and prognostic stratification.

Keyword co-occurrence analysis showed that major terms were interconnected rather than distributed as isolated lexical units. Inflammatory pathways, immune regulation, biomarker discovery, prognostic assessment, and organ dysfunction were closely linked, indicating that host response-oriented sepsis research has developed around overlapping mechanistic and translational modules (**Figure 7**).

**Figure 8:**
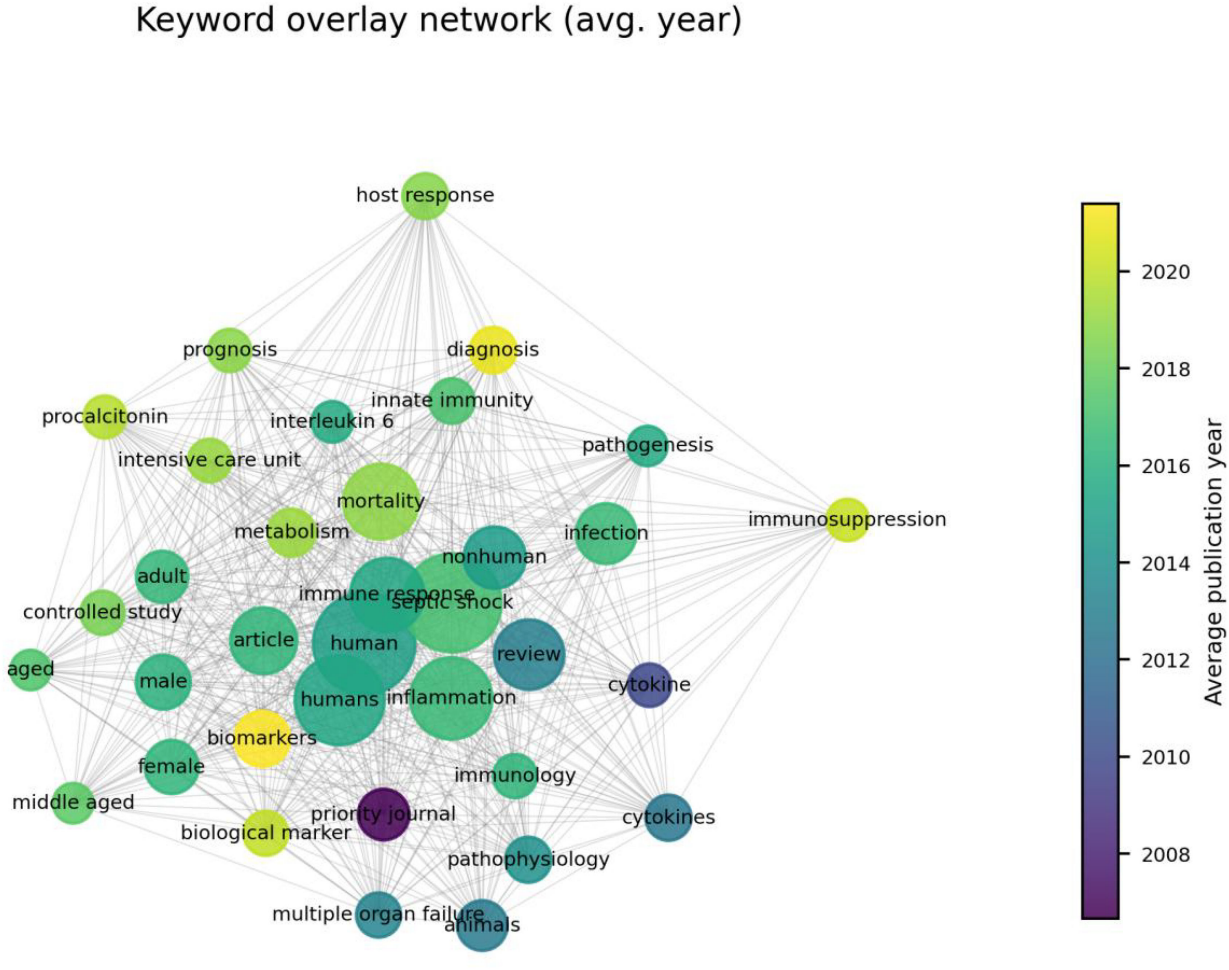
Keyword overlay network of host response-oriented sepsis research. The overlay network visualizes the temporal distribution of high-frequency keywords. Node size indicates keyword frequency, and node color represents the average publication year of documents containing each keyword. The image highlights the shift from classical inflammatory and immune activation themes toward biomarkers, molecular stratification, prognostic assessment, and precision-oriented research.

The keyword overlay map further showed how research attention changed over time. Earlier terms were more strongly associated with inflammatory activation, LPS signaling, cytokine pathways, and classical immune mechanisms, whereas more recent terms were concentrated around biomarkers, prognostic evaluation, molecular stratification, and precision-oriented identification (**Figure 8**).

### Selection of the optimal topic solution

Across K = 4 to 12 solutions, c_v coherence ranged from 0.469 to 0.527, peaking at K = 12 (0.5272) with a local maximum at K = 8 (0.5184); seed stability was highest at K = 4 (0.4893) and fell below 0.33 for K of 9 or more, while the minimum topic size declined from 422 (K = 4) to 47 (K = 12). The eight-topic model offered the best overall balance among coherence, stability, topic-size balance, and interpretability. In lower-dimensional solutions several research directions remained overly broad, whereas at higher topic numbers the solutions became progressively fragmented; the eight-topic model separated inflammatory and innate-immune signaling, transcriptomics and gene expression, diagnostic and prognostic biomarkers, clinical management and precision medicine, organ dysfunction, ICU outcomes, sepsis definitions, and a distinct viral/parasitic host-response domain (**Supplementary Table 3**).

### The eight thematic domains of host response-oriented sepsis research

The final eight-topic LDA model identified multiple thematic domains rather than a single dominant research direction. As summarized in **Table 1**, the identified domains covered inflammatory and innate-immune signaling; transcriptomics and gene expression; diagnostic and prognostic biomarkers; clinical management, therapeutic advances, and precision medicine; organ dysfunction, endothelial injury, and coagulation; ICU outcomes and mortality risk; sepsis definitions and early management; and viral and parasitic host-response studies. The largest domains were Inflammatory Responses, LPS/Innate Immune Signaling, and Cytokine Pathways (n=768, 25.8%), Clinical Management, Therapeutic Advances, and Precision Medicine (n=573, 19.3%), and Organ Dysfunction, Endothelial Injury, and Coagulation (n=392, 13.2%). The basis for topic labeling and representative publications is further provided in **Supplementary Table 2**.

**Table 1:** The eight LDA-defined thematic domains, publication sizes, and thematic interpretations.

Topic No.	Thematic domain	Publications	Proportion	Selected representative model terms	Thematic interpretation
1	Inflammatory Responses, LPS/Innate Immune Signaling, and Cytokine Pathways	768	25.82%	inflammatory response; immune activation; LPS signaling; Toll-like receptors; cytokines; innate immunity; bacterial recognition	This domain captures inflammatory activation, LPS and Toll-like-receptor signaling, cytokine pathways, and innate immune responses.
2	Transcriptomics, Gene Expression, and Bioinformatic Profiling	327	11.00%	gene expression; transcriptomics; RNA sequencing; non-coding RNA; bioinformatics; molecular features	This domain reflects gene-expression profiling, RNA sequencing, transcriptomic signatures, and bioinformatic identification of molecular features.
3	Diagnostic and Prognostic Biomarkers (Procalcitonin and Severity Scores)	267	8.98%	biomarkers; procalcitonin; diagnosis; prognosis; severity scores; SOFA; biomarker levels	This domain focuses on diagnostic and prognostic biomarkers, procalcitonin, and severity scores for outcome assessment.
4	Clinical Management, Therapeutic Advances, and Precision Medicine	573	19.27%	treatment; therapy; clinical management; therapeutic advances; precision medicine; antimicrobial therapy	This domain includes therapeutic advances, clinical management, antimicrobial therapy, and precision or omics-guided medicine.
5	Organ Dysfunction, Endothelial Injury, and Coagulation	392	13.18%	organ dysfunction; injury; endothelial; coagulation; complement; systemic; mitochondrial	This domain centers on organ dysfunction, endothelial injury, coagulation, complement, and systemic injury mechanisms.
6	ICU Outcomes, Mortality Risk, and Critical Illness Complications	364	12.24%	mortality; ICU; risk; pneumonia; critically ill; intensive care; outcomes	This domain links ICU outcomes, mortality risk, pneumonia, critical illness, and ICU-acquired complications.
7	Sepsis Definitions, Diagnostic Criteria, and Early Management	208	6.99%	criteria; SIRS; Sepsis-3; definitions; early management; resuscitation; fluid	This domain focuses on sepsis definitions and diagnostic criteria (SIRS and Sepsis-3), early management, and resuscitation.
8	Viral and Parasitic Infections and Host Response (HIV, Hepatitis, Malaria)	75	2.52%	virus; viral; HIV; hepatitis; malaria; viremia; influenza	This domain comprises viral and parasitic host-response studies, including HIV, hepatitis, malaria, and influenza, and viremia.

Topic labels were assigned by author-team consensus based on high-weight model terms, representative publications with high topic probabilities, and semantic coherence. Publication counts reflect dominant topic assignment based on the maximum document-topic posterior probability from the final K = 8 LDA model; proportions used the final analyzed corpus (2,974 records) as the denominator. Selected representative model terms were curated from high-weight model terms to improve readability while preserving the interpretation of the original LDA-derived themes.

To further document the influential knowledge base captured by the search strategy, highly cited publications are summarized in **Table 2**.

**Table 2:** Highly cited publications identified in the final bibliometric dataset.

Title	Year	Journal	DOI	Source databases	Citation count
The third international consensus definitions for sepsis and septic shock (sepsis-3)	2016	JAMA - Journal of the American Medical Association	10.1001/jama.2016.0287	Scopus; WoSCC	20047
Global, regional, and national sepsis incidence and mortality, 1990-2017: analysis for the Global Burden of Disease Study	2020	LANCET	10.1016/S0140-6736(19)32989-7	WoSCC	4778
Assessment of clinical criteria for sepsis for the third international consensus definitions for sepsis and septic shock (sepsis-3)	2016	JAMA - Journal of the American Medical Association	10.1001/jama.2016.0288	Scopus; WoSCC	2830
The immunopathogenesis of sepsis	2002	Nature	10.1038/nature01326	Scopus; WoSCC	2198
Sepsis-induced immunosuppression: from cellular dysfunctions to immunotherapy	2013	NATURE REVIEWS IMMUNOLOGY	10.1038/nri3552	WoSCC	2037
The immunopathology of sepsis and potential therapeutic targets	2017	Nature Reviews Immunology	10.1038/nri.2017.36	Scopus; WoSCC	1452
Invasive candidiasis	2018	Nature Reviews Disease Primers	10.1038/nrdp.2018.26	Scopus; WoSCC	1209
Cytokine storm and sepsis disease pathogenesis	2017	Seminars in Immunopathology	10.1007/s00281-017-0639-8	Scopus; WoSCC	1018
Derivation, Validation, and Potential Treatment Implications of Novel Clinical Phenotypes for Sepsis	2019	JAMA - Journal of the American Medical Association	10.1001/jama.2019.5791	Scopus; WoSCC	979
Harmful molecular mechanisms in sepsis	2008	NATURE REVIEWS IMMUNOLOGY	10.1038/nri2402	WoSCC	968
The role of the endothelium in severe sepsis and multiple organ dysfunction syndrome	2003	Blood	10.1182/blood-2002-06-1887	Scopus; WoSCC	910
Enhancing recovery from sepsis: A review	2018	JAMA - Journal of the American Medical Association	10.1001/jama.2017.17687	Scopus; WoSCC	774
Advances in the understanding and treatment of sepsis-induced immunosuppression	2018	Nature Reviews Nephrology	10.1038/nrneph.2017.165	Scopus; WoSCC	732
A 3-level prognostic classification in septic shock based on cortisol levels and cortisol response to corticotropin	2000	JAMA	10.1001/jama.283.8.1038	Scopus; WoSCC	719
The immunology of sepsis	2021	Immunity	10.1016/j.immuni.2021.10.012	Scopus; WoSCC	688
Hydrocortisone plus fludrocortisone for adults with septic shock	2018	New England Journal of Medicine	10.1056/NEJMoa1705716	Scopus; WoSCC	677
COVID-19: immunopathogenesis and Immunotherapeutics	2020	Signal Transduction and Targeted Therapy	10.1038/s41392-020-00243-2	Scopus; WoSCC	615
Genomic landscape of the individual host response and outcomes in sepsis: A prospective cohort study	2016	The Lancet Respiratory Medicine	10.1016/S2213-2600(16)00046-1	Scopus; WoSCC	601
Cytokines in Sepsis: Potent Immunoregulators and Potential Therapeutic Targets-An Updated View	2013	MEDIATORS OF INFLAMMATION	10.1155/2013/165974	WoSCC	582

Citation counts were derived from citation fields available in the original bibliographic exports and reflect the values captured at the final search/export stage rather than live citation totals. For records present in multiple databases, citation values may differ across sources; therefore, this table should be interpreted as a descriptive indicator of the influential knowledge base captured by the search strategy rather than as a formally normalized citation analysis.

**Figure 9:**
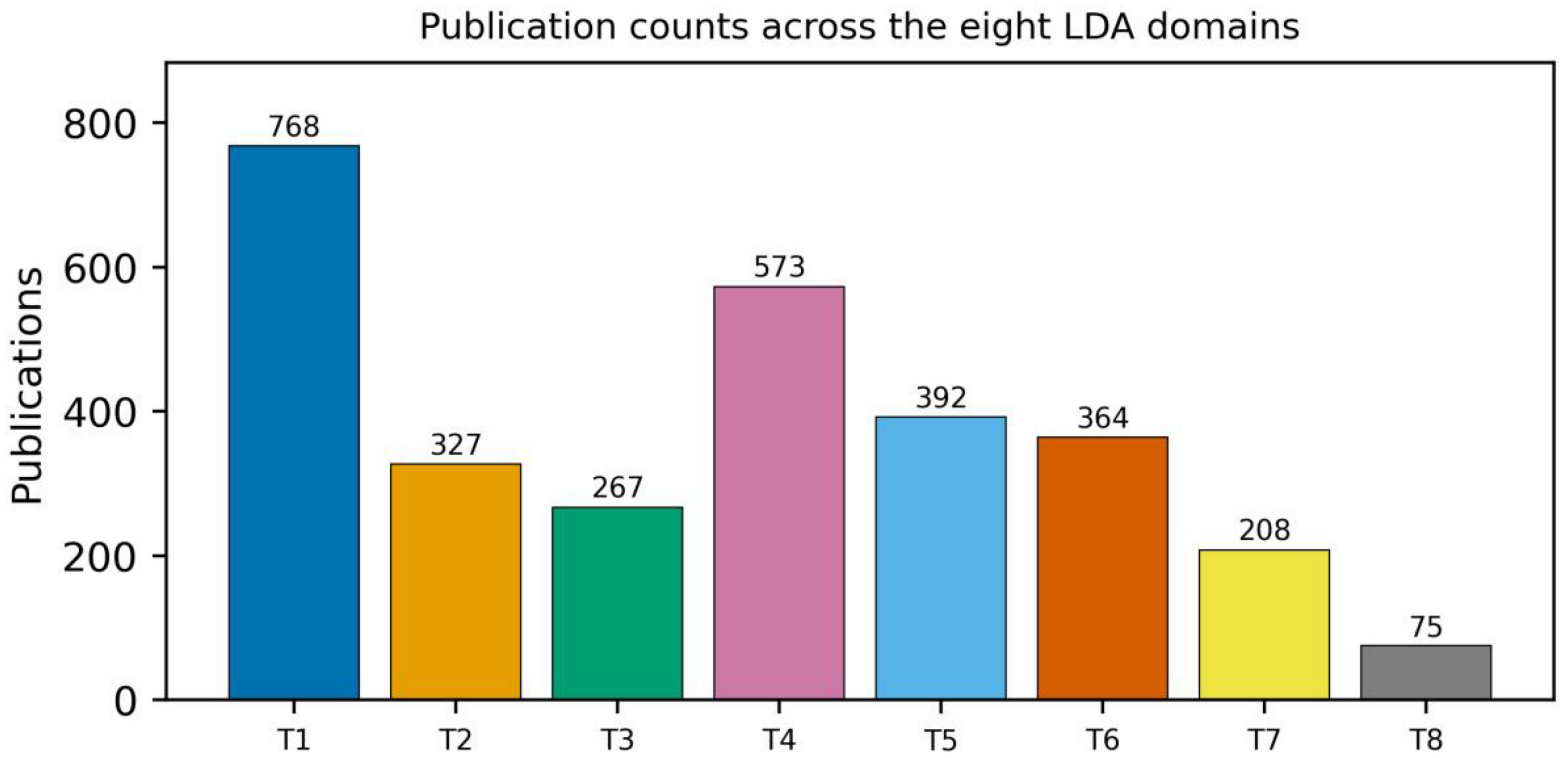
Publication counts across the eight LDA-defined thematic domains. Each publication was assigned to its dominant topic according to the highest posterior topic probability. Topic size represents the number of publications assigned to each thematic domain. The image illustrates the relative weight of the eight domains within the overall knowledge structure of host response-oriented sepsis research.

The distribution of dominant topic assignments showed that inflammatory and innate-immune signaling, clinical management and precision medicine, and organ dysfunction constituted the largest thematic domains (**Figure 9**). Inflammatory signaling and clinical management represented long-standing core areas, whereas transcriptomics, diagnostic and prognostic biomarkers, and ICU outcome themes showed a stronger translational orientation. The viral and parasitic host-response domain remained small and should be interpreted cautiously. Because this topic included boundary infection-related host-response records, especially viral and parasitic studies, it should be interpreted as an adjacent host-response domain captured by the search strategy rather than as a core bacterial sepsis domain.

### Temporal evolution of the identified topics

**Figure 10:**
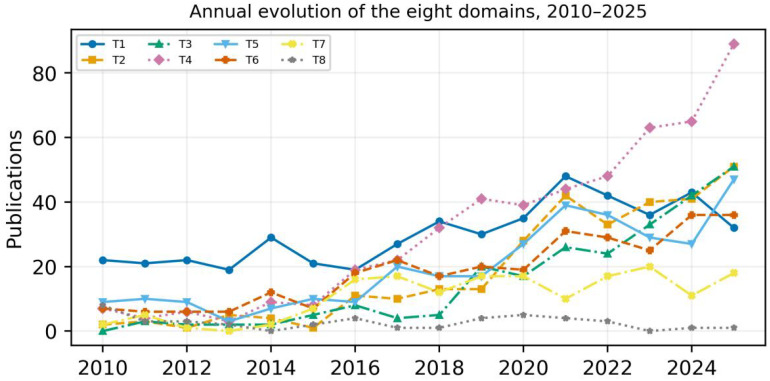
Annual evolution of the eight LDA-defined thematic domains, 2010–2025. Annual topic-specific publication counts were calculated according to dominant topic assignment. The image shows the expansion, differentiation, and relative temporal changes of the eight thematic domains during the contemporary phase of host response-oriented sepsis research.

Annual topic-specific publication counts from 2010 to 2025 were calculated to compare temporal patterns across the eight domains. The resulting trend curves showed that inflammatory and innate-immune signaling and clinical management remained long-standing foundational themes, whereas transcriptomics, diagnostic and prognostic biomarkers, and ICU outcome and prognostic themes showed apparent recent increases at the macro-thematic level (**Figure 10**). The main trend analysis focused on 2010–2025 to highlight the contemporary growth phase, whereas the full 2000–2025 topic evolution is provided in **Supplementary Figure 1**. The annual topic-count matrix is listed in **Supplementary Table 5**.

Because these annual curves are based on absolute publication counts and total output increased over time, temporal differences should be interpreted as descriptive shifts in thematic emphasis rather than as normalized incidence rates. In the annual topic-count matrix, Topic 1 changed from 19/104 records in 2016 to 32/325 records in 2025, whereas Topic 2 increased from 11/104 to 51/325 and Topic 4 increased from 19/104 to 89/325. These descriptive proportions support a shift in relative thematic emphasis, although the exact topic boundaries remain preprocessing-dependent.

**Figure 11:**
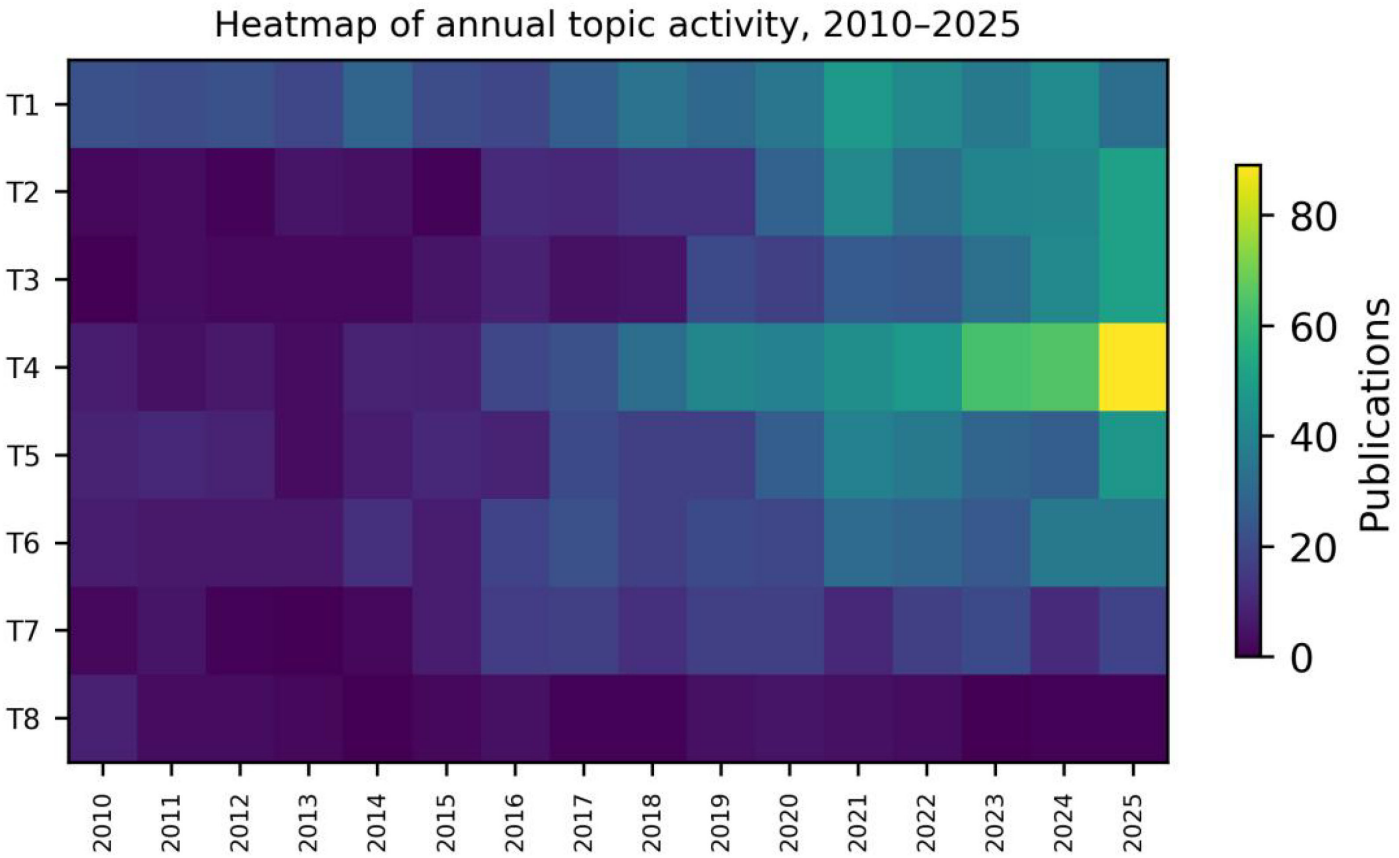
Heatmap of annual topic activity, 2010–2025. The heatmap presents annual publication activity for each LDA-defined thematic domain. Darker color intensity indicates higher publication activity for a given topic-year combination. This visualization provides an integrated overview of temporal differences among the eight domains and supports the interpretation of recent thematic shifts toward molecular characterization and precision stratification.

The topic activity heatmap provided an additional view of year-specific topic activity across the eight domains. It was consistent with the persistent activity of inflammatory and innate-immune signaling and clinical-management themes and suggested apparent recent increases in transcriptomics, diagnostic and prognostic biomarkers, and ICU outcome and prognostic research (**Figure 11**). Together, the thematic structure, keyword overlay pattern, and temporal evolution results suggest a gradual shift from classical inflammatory mechanisms toward molecular characterization, precision stratification, and individualized clinical management.

## Discussion

### Principal findings

The present analysis indicates that host response-oriented sepsis research is not limited to inflammation-centered mechanisms but now spans multiple mechanistic, diagnostic, prognostic, and translational domains (9, 10). Beyond describing this trajectory, the added value of this study lies in its focused, adult-oriented, multi-database corpus integrating Web of Science Core Collection, Scopus, and PubMed; its DOI plus metadata-adjudicated normalized-title deduplication, which avoids over-merging generic-title literature; its assignment of each publication to a dominant topic from the estimated document-topic posterior probability; and its coupling of thematic structure with annual evolution to identify future research priorities rather than merely restating topic proportions (4, 22). The main novelty of this study therefore lies in its host-response-specific corpus, strict three-database deduplication strategy, LDA-derived thematic structure, temporal evolution analysis, and transparent sensitivity analyses, which together provide a reproducible overview of how this focused sepsis subfield has evolved over time.

A second major finding is the apparent recent increase, in absolute publication counts and at the macro-thematic level, of biomarker-, transcriptomics-, and precision-stratification-related themes (11, 12). These domains showed apparent increases in absolute publication counts and appear to represent active areas of current research, although their exact boundaries remain preprocessing-dependent.

### Interpretation of the findings

The observed transition from classical inflammatory mechanisms to molecular characterization and precision stratification reflects a broader shift in sepsis research (23, 24). Earlier work primarily sought to clarify the basic pathophysiology of host response after infection (25, 26). As the limitations of one-size-fits-all approaches became more evident, attention progressively shifted toward identifying biologically meaningful subgroups, risk trajectories, and treatment-relevant host-response states.

Host-response heterogeneity now provides a practical link between mechanistic studies and clinically oriented stratification strategies (7, 27). Studies of immune phenotypes, endotypes, transcriptomic signatures, and multimarker panels are increasingly directed toward early recognition, prognostic evaluation, subtype identification, and individualized therapeutic decision support (12, 28).

These findings should not be interpreted as a simple linear progression from inflammation research to precision medicine. Rather, they suggest a field in which classical inflammatory biology, biomarker discovery, transcriptomic endotyping, and clinical-management research are expanding at different speeds and are not yet fully integrated. The key unresolved issue is whether the rapidly growing molecular and biomarker domains can modify clinical decision-making beyond descriptive subgroup identification. Therefore, the major future challenge is to convert thematic growth into externally validated, time-sensitive, and clinically actionable stratification frameworks.

### Relation to previous studies

Compared with general bibliometric analyses of sepsis, this study focuses specifically on the host-response perspective and therefore provides a more focused view of a precision-oriented research subfield (15). In addition, whereas keyword-based mapping mainly reflects explicit lexical relationships, LDA helps identify latent thematic structures that may not be fully captured by co-occurrence analysis alone (16, 18).

The present study also adds methodological value through strict cross-database deduplication and explicit temporal evolution analysis (18, 19). These procedures reduced duplicate records and improved the interpretability of the topic structure.

### Methodological significance

Methodologically, this study illustrates the value of combining bibliometric analysis, topic modeling, and temporal evolution analysis within a single workflow (16, 18). Bibliometric methods summarize production patterns, collaboration structures, journal dissemination, and keyword networks, whereas LDA captures latent semantic structures that are not fully accessible through keyword co-occurrence alone.

Strict cross-database deduplication further improved data reliability by reducing bibliographic redundancy before topic modeling.

### Practical implications

The thematic evolution identified in this study suggests several concrete priorities for the next stage of host response-oriented sepsis research. First, the parallel recent expansion of Topic 2 (transcriptomics, gene expression, and bioinformatic profiling) and Topic 3 (diagnostic and prognostic biomarkers) indicates that molecular endotyping and clinically deployable biomarker assessment have developed as active but partly separated research streams (29, 30). A priority is therefore not simply to generate additional signatures, but to prospectively test whether transcriptomic endotypes can be translated into parsimonious multimarker panels that retain prognostic and treatment-stratification value across cohorts, infection sources, and sampling windows (31).

Second, the contrast between the persistent but relatively less expanding Topic 7 (sepsis definitions, diagnostic criteria, and early management) and the rapid growth of Topic 4 (clinical management, therapeutic advances, and precision medicine) highlights a translational gap. Sepsis-3 and early-management frameworks remain clinically dominant, but they are not yet routinely linked to molecular or immune-response stratification. Future studies should therefore evaluate whether host-response endotypes can be embedded into clinically timed decision points, such as early risk triage, antimicrobial escalation or de-escalation, immunomodulatory trial enrichment, and prediction of organ-support trajectories.

Third, Topic 1 and Topic 5 show that inflammatory signaling, innate immunity, organ dysfunction, endothelial injury, and coagulation remain foundational knowledge domains, but their clinical translation remains incomplete. The next priority is to move from isolated pathway description toward longitudinal host-response trajectories that connect immune activation or suppression with endothelial dysfunction, coagulation disturbance, and organ-failure progression. Such designs would better support dynamic risk stratification than single-time-point biomarker studies.

Finally, the small viral and parasitic host-response domain indicates that broader infection-related host-response literature enters the corpus at the boundary of bacterial sepsis research. This suggests that future reviews and validation studies should explicitly distinguish generalizable host-response mechanisms from pathogen-class-specific patterns, especially when post-2020 transcriptomic expansion may partly reflect COVID-19-related host-response studies.

### Limitations

Several limitations should be acknowledged. First, the dataset was restricted to publications indexed in Web of Science Core Collection, Scopus, and PubMed, and non-English or grey literature may not be fully covered. Second, neonatal, pediatric, and animal-related records were excluded at the retrieval stage; this adult-focused scope was deliberate but limits generalizability to pediatric, neonatal, and experimental host-response biology. Third, although journal, country, institution, and author metadata were normalized, author-name ambiguity could not be fully resolved, and abbreviated-name aggregates such as Wang Y and Zhang Y represent standardized strings rather than confirmed single individuals. Fourth, LDA results depend on corpus definition, preprocessing, vocabulary filtering, and the choice of K; preprocessing sensitivity analyses showed that only the broad macro-level domains were partly reproducible (top-20 term overlap 0.10 to 0.44; dominant-topic agreement 0.41 to 0.56 with the primary pipeline), so the fine-grained structure is preprocessing-dependent and the topics should be interpreted as exploratory thematic structures rather than fixed categories. Fifth, assigning each publication to a single dominant topic may underrepresent highly interdisciplinary studies; the full posterior is therefore retained so that small-margin documents are transparent. In addition, the post-2020 expansion of transcriptomics and host-response profiling may have been partly influenced by COVID-19-related literature and pandemic-era interest in viral host-response biology. Therefore, recent increases in transcriptomic and molecular profiling themes should not be interpreted as arising exclusively from non-COVID adult sepsis research. The small viral and parasitic host-response domain should also be interpreted cautiously. Some high-probability records in this topic reflect boundary or adjacent infection-related host-response literature rather than classical adult bacterial sepsis, indicating that the search strategy captured a limited amount of broader host-response literature at the viral/parasitic infection interface. Finally, bibliometric and topic-modeling analyses describe publication patterns and semantic structures, but they do not assess the methodological quality or clinical validity of individual studies.

## Conclusion

This study mapped the knowledge structure and temporal evolution of host response-oriented sepsis research using an integrated bibliometric and LDA-based approach, showing that the literature is organized around several distinct thematic domains and provides descriptive evidence of a shift in publication activity from an inflammation-centered paradigm toward molecular characterization, precision stratification, and individualized management.

Transcriptomics, molecular biomarkers, prognostic stratification, and early diagnostic strategies are likely to remain central topics for future reviews, study design, and identification of emerging research priorities.

## Data Availability

The original contributions presented in the study are included in the article/**Supplementary Material**. Further inquiries can be directed to the corresponding author.
