# Supplementary material for: Knowledge structure and thematic evolution of host response-oriented sepsis research: a multi-database bibliometric and LDA topic modeling study
Source: Front Immunol. 2026 Jul 1;17:1872111. doi: 10.3389/fimmu.2026.1872111 (PMC13368975; doi:10.3389/fimmu.2026.1872111)
Supplement: Supplementary file 1 [file DataSheet1.docx]

# **Supplementary Material**

**Knowledge Structure and Thematic Evolution of Host Response-Oriented Sepsis Research: A Multi-Database Bibliometric and LDA Topic Modeling Study**

This Supplementary Material contains the Supplementary Methods, Supplementary Tables 1-8, and Supplementary Figures 1-3 supporting the main text. All quantitative values were derived from the final analyzed corpus (N = 2,974) and the final K = 8 latent Dirichlet allocation (LDA) model; topic assignments are the dominant topic based on the maximum document-topic posterior probability.

## Supplementary Methods

### **S1. Data sources and structured search strategy**

Bibliographic records were retrieved from PubMed, Scopus, and the Web of Science Core Collection and combined into a single dataset. The search combined a sepsis axis with a host-response axis and excluded neonatal, pediatric, and animal-related records at the retrieval stage. The final search date was 29 March 2026. Database-specific strings are in Supplementary Table 1.

The database-specific strings are reported as executed using the syntax available in each platform. Minor differences in wildcarding, field tags, and phrase nesting reflect platform-specific search syntax and were preserved for transparency. Therefore, the record counts and downstream analyses correspond to the executed database-specific searches reported in Supplementary Table 1 rather than to a post hoc harmonized query.

### **S2. Data integration and metadata-adjudicated deduplication**

Two records without titles were removed before deduplication. Duplicates were then removed in two stages: (i) DOI matching after normalization (lowercasing; removal of resolver and doi prefixes; trimming of trailing punctuation; empty DOIs unused); and (ii) metadata-adjudicated normalized-title matching. Within each identical-normalized-title group, two records were treated as the same publication only if (A1) their DOIs were equivalent after format normalization (case, hyphen/underscore, leading zeros), or (A2) they shared the same first-author surname and publication year; otherwise they were retained as distinct works. This removed 2,789 records by DOI matching and 74 records by adjudicated title matching, yielding 2,974 unique publications. Per-group decisions are in Supplementary Table 8. Because identical normalized titles may occur in records with different DOI strings, the number of distinct normalized DOIs was retained in Supplementary Table 8 for transparency. The A2 rule was applied only within identical-normalized-title groups sharing the same first-author surname and publication year; generic titles or groups not satisfying the predefined criteria were retained as distinct works. These decisions were reported at the group level to make boundary cases auditable.

### **S3. Corpus construction, LDA, and topic assignment**

Title, abstract, author keywords, and index keywords were concatenated; text was lowercased; URLs, non-alphabetic symbols, and redundant whitespace were removed; standard English stopwords, the query terms sepsis/septic/shock, and demographic MeSH/check-tag noise terms were removed; tokens shorter than three characters were discarded; lemmatization was not applied (primary pipeline). Terms in fewer than 5 documents (no_below = 5) or more than 50% of documents (no_above = 0.5) were filtered, giving a 2,974 x 6,083 document-term matrix. LDA was implemented in Python using the LdaModel class from gensim (version 4.4.0); K = 4-12 were fitted across seeds 42, 7, 2026 (passes = 20, iterations = 200, alpha = auto, eta = auto). The final K = 8 model used random_state = 42, and each publication was assigned to its dominant topic (maximum posterior probability).

### **S4. Metadata normalization**

Journal titles were standardized by case normalization, abbreviation harmonization, and variant-to-canonical mapping (e.g., Shock / SHOCK / Shock (Augusta, Ga.) -> Shock). Country names were harmonized (e.g., USA / United States of America -> United States). Institution names were harmonized using representative variant-to-canonical mappings. Author names were standardized to surname-initial format; common surname-initial strings such as Wang Y and Zhang Y were treated cautiously as potentially ambiguous aggregates. Web of Science continuation-line author parsing was corrected and verified. Full counting was used unless otherwise specified.

## **Supplementary Table 1. Database-specific search strategies and final search date.**

| Database | Search fields | Final search date | Records | Complete search strategy |
| --- | --- | --- | --- | --- |
| PubMed | Title/Abstract + MeSH | 29 March 2026 | 1,823 | (“Sepsis”[Mesh] OR “Shock, Septic”[Mesh] OR sepsis[tiab] OR “septic shock”[tiab]) AND (“host response”[tiab] OR “dysregulated host response”[tiab] OR “host immune response”[tiab] OR “immune phenotype”[tiab] OR “immune endotype”[tiab] OR endotype[tiab] OR ((“biomarker panel”[tiab] OR multimarker[tiab] OR “multi-marker”[tiab] OR “multi marker”[tiab]) AND (stratif[tiab] OR phenotype[tiab] OR endotype[tiab] OR “host response”[tiab]))) NOT (neonat[tiab] OR pediatric[tiab] OR paediatric[tiab] OR child[tiab] OR infant[tiab] OR mouse[tiab] OR mice[tiab] OR murine[tiab] OR rat[tiab] OR rats[tiab] OR dog[tiab] OR dogs[tiab] OR cat[tiab] OR cats[tiab] OR pig[tiab] OR pigs[tiab] OR swine[tiab] OR porcine[tiab] OR canine[tiab] OR feline[tiab] OR sheep[tiab] OR ovine[tiab] OR horse[tiab] OR horses[tiab] OR equine[tiab] OR rabbit[tiab] OR rabbits[tiab]) |
| Web of Science Core Collection | Topic (TI, AB, author keywords, Keywords Plus) | 29 March 2026 | 1,636 | (sepsis OR “septic shock”) AND (“host response” OR “dysregulated host response” OR “host immune response” OR “immune phenotype” OR “immune endotype” OR endotype* OR ((“biomarker panel” OR multimarker* OR “multi-marker” OR “multi marker”) AND (stratif* OR phenotype* OR endotype* OR “host response”))) NOT (neonat* OR pediatric* OR paediatric* OR child* OR infant* OR mouse OR mice OR murine OR rat OR rats OR dog OR dogs OR cat OR cats OR pig OR pigs OR swine OR porcine OR canine OR feline OR sheep OR ovine OR horse OR horses OR equine OR rabbit OR rabbits) |
| Scopus | TITLE-ABS-KEY | 29 March 2026 | 2,380 | TITLE-ABS-KEY(sepsis OR “septic shock”) AND TITLE-ABS-KEY(“host response” OR “dysregulated host response” OR “host immune response” OR “immune phenotype” OR “immune endotype” OR endotype* OR ((“biomarker panel” OR multimarker* OR “multi-marker” OR “multi marker”) AND (stratif* OR phenotype* OR endotype))) AND NOT TITLE-ABS-KEY(neonat OR pediatric* OR paediatric* OR child* OR infant* OR mouse OR mice OR murine OR rat OR rats OR dog OR dogs OR cat OR cats OR pig OR pigs OR swine OR porcine OR canine OR feline OR sheep OR ovine OR horse OR horses OR equine OR rabbit OR rabbits) |
| Total | — | 29 March 2026 | 5,839 | PubMed + Scopus + WoSCC before screening |

Inclusion: sepsis/septic shock combined with host response, immune phenotype/endotype, and biomarker/multimarker stratification concepts. Exclusion at retrieval: neonatal, pediatric, and animal terms.

Search strings are reported exactly as executed in each database. The Scopus query was not retrospectively harmonized for wildcard use; therefore, terms such as neonat and endotype are preserved as executed. The reported record counts and all downstream analyses correspond to the executed searches rather than to a post hoc harmonized search strategy.

## **Supplementary Table 2. Topic-labeling support for the eight LDA-defined thematic domains.**

| Topic No. | Final thematic label | High-weight model terms | Representative publications (high topic probability) | Interpretation rationale |
| --- | --- | --- | --- | --- |
| 1 | Inflammatory Responses, LPS/Innate Immune Signaling, and Cytokine Pathways | cell; protein; cells; factor; receptor; inflammatory; bacterial; interleukin; cytokine; inflammation; like; innate | Co-operation of TLR4 and raft proteins in LPS-induced pro-inflammatory signaling; Innate immune responses to infection; Bacterial recognition and induced cell activation in sepsis; Macrophage CD40 signaling: A pivotal regulator of disease protection and pathogenesis | High-weight terms (inflammatory, cytokine, lps, receptor, toll, innate, interleukin) and TLR4/LPS-signaling representatives. |
| 2 | Transcriptomics, Gene Expression, and Bioinformatic Profiling | gene; expression; analysis; covid; genes; rna; cell; identified; study; using; data; blood | Blood transcriptome analysis reveals CTSB and ATP6V0D1 expression in circulating monocytes as potential biomarkers of sepsis; Dysregulated lncRNAs are Involved in the Progress of Sepsis by Constructing Regulatory Networks in Whole Blood Cells; PAGE-based transfer learning from single-cell to bulk sequencing enhances model generalization for sepsis diagnosis; Identification of the TF-miRNA-mRNA co-regulatory networks involved in sepsis | High-weight terms (gene, expression, rna, covid, transcriptome, profiling) and RNA-seq/bioinformatics representatives. |
| 3 | Diagnostic and Prognostic Biomarkers (Procalcitonin and Severity Scores) | study; mortality; biomarkers; blood; score; diagnosis; procalcitonin; diagnostic; prognosis; protein; analysis; pct | Clinical Predictive Value of Immature Platelet Fraction Combined with Other Biomarkers for the Severity and Prognosis of Sepsis; Analysis of the D-dimer/Platelets Ratio in Sepsis Patients at Dr. Wahidin Sudirohusodo Hospital Makassar; Relationship between Protein C and Antithrombin Levels with SOFA Score in Sepsis; Serum Procalcitonin vs SOFA Score in Predicting Outcome in Sepsis Patients in Medical Intensive Care Unit | High-weight terms (biomarkers, procalcitonin, pct, score, diagnosis, prognosis) and SOFA/biomarker representatives. |
| 4 | Clinical Management, Therapeutic Advances, and Precision Medicine | treatment; review; care; mortality; therapy; organ; research; medicine; dysfunction; management; disease; diagnosis | Sepsis biomarkers: past, present, and future; The Clinical View of Sepsis-Associated AKI: How Basic Science Can Help Solve This Problem; Precision medicine in sepsis and septic shock: From omics to clinical tools; The evolution of antimicrobial therapy for sepsis | High-weight terms (treatment, therapy, management, therapeutic, precision, medicine) and review/precision-medicine representatives. |
| 5 | Organ Dysfunction, Endothelial Injury, and Coagulation | dysfunction; organ; inflammatory; injury; inflammation; failure; endothelial; coagulation; systemic; multiple; review; system | Free Radicals, Mitochondrial Dysfunction and Sepsis-induced Organ Dysfunction: A Mechanistic Insight; Complement system; Platelet activation and antiplatelet therapy in sepsis: A narrative review; Sepsis-Induced Myocardial Dysfunction (SIMD): the Pathophysiological Mechanisms and Therapeutic Strategies Targeting Mitochondria | High-weight terms (dysfunction, organ, injury, endothelial, coagulation, systemic) and organ-injury-mechanism representatives. |
| 6 | ICU Outcomes, Mortality Risk, and Critical Illness Complications | mortality; study; associated; icu; levels; risk; care; day; pneumonia; results; blood; critically | Patients with faecal peritonitis admitted to European intensive care units: an epidemiological survey of the GenOSept cohort; Mortality and host response aberrations associated with transient and persistent acute kidney injury in critically ill patients with sepsis: a prospective cohort study; Plasma protein biomarkers reflective of the host response in patients developing Intensive Care Unit-acquired pneumonia; The Host Response in Patients with Sepsis Developing Intensive Care Unit-acquired Secondary Infections | High-weight terms (mortality, icu, risk, pneumonia, critically, ill) and ICU-outcome/critical-illness representatives. |
| 7 | Sepsis Definitions, Diagnostic Criteria, and Early Management | organ; therapy; care; dysfunction; mortality; early; failure; treatment; resuscitation; criteria; sirs; fluid | Are you Ernest Shackleton, the polar explorer? Refining the criteria for delirium and brain dysfunction in sepsis; Hospital and intensive care unit admission criteria for the septic patient; Sepsis and Septic Shock; S3 Guideline: Sepsis 2018 | High-weight terms (criteria, sirs, resuscitation, early, fluid, definitions) and Sepsis-3/guideline representatives. |
| 8 | Viral and Parasitic Infections and Host Response (HIV, Hepatitis, Malaria) | virus; viral; liver; disease; hiv; infections; malaria; infected; viremia; fever; hepatitis; influenza | Hepatitis B surface antigen: relation to hepatitis B replication parameters in HBeAg-negative chronic hepatitis B; Hepatic fibrosis and immune phenotype vary by HCV viremia in HCV/HIV co-infected subjects; Immune response to Plasmodium vivax has a potential to reduce malaria severity; Kinetics of hepatitis C virus load and hemodialysis: is there any influence of the reuse of dialysis membrane on HCV viremia? | High-weight terms (virus, viral, hiv, hepatitis, malaria, viremia) and viral/parasitic-infection representatives. |

High-weight terms are the top model terms per topic; representative publications are the highest document-topic-probability titles; interpretation rationales summarize the semantic basis for each label.

## **Supplementary Table 3. Topic-number diagnostics for K = 4-12 (gensim LDA; seeds 42, 7, 2026).**

| K | c_v coherence (mean) | Log-perplexity (mean) | Top-20 Jaccard stability (mean) | Min. topic size (mean across seeds) | Selection note |
| --- | --- | --- | --- | --- | --- |
| 4 | 0.4817 | -7.4428 | 0.4893 | 421.7 | highest stability |
| 5 | 0.4687 | -7.4263 | 0.3737 | 179.7 | — |
| 6 | 0.4973 | -7.4149 | 0.4251 | 137.0 | — |
| 7 | 0.4966 | -7.4071 | 0.403 | 117.3 | — |
| 8 | 0.5184 | -7.4043 | 0.3775 | 83.0 | selected (balanced compromise) |
| 9 | 0.5119 | -7.3994 | 0.3289 | 85.7 | — |
| 10 | 0.5112 | -7.3981 | 0.3228 | 67.3 | — |
| 11 | 0.5234 | -7.3901 | 0.3203 | 60.0 | — |
| 12 | 0.5272 | -7.3903 | 0.3081 | 47.3 | highest coherence |

K = 8 was selected as a balanced compromise considering coherence, stability, topic-size balance, and interpretability, not the best model on every metric (highest c_v at K = 12; highest seed stability at K = 4). All diagnostics are means across the three seeds at the model-selection stage. The minimum topic size here (e.g., 83 at K = 8) is the seed-level model-selection diagnostic; the final dominant-topic counts reported in main-text Table 1 (e.g., 75 for Topic 8) are from the selected random_state = 42 model. The K = 8 c_v reported here (0.5184, seed mean) differs slightly from the single-model primary-pipeline value in Supplementary Table 7 (0.5156) for the same reason.

## **Supplementary Table 4. Representative metadata normalization mappings and normalized bibliometric rankings.**

**Panel A. Representative variant-to-canonical mappings**

| Field | Representative variants | Canonical form |
| --- | --- | --- |
| Journal | SHOCK; Shock; Shock (Augusta, Ga.) | Shock |
| Journal | Frontiers in Immunology; FRONTIERS IN IMMUNOLOGY | Frontiers in Immunology |
| Country | USA; United States; U.S.A.; United States of America | United States |
| Country | England; Scotland; Wales; UK; United Kingdom | United Kingdom |
| Country | Peoples R China; China; PR China | China |
| Institution | University of Amsterdam; Amsterdam UMC; Academic Medical Center | University of Amsterdam / Amsterdam UMC |
| Author | Van Der Poll T (full: van der Poll, Tom) | Van Der Poll T (not ambiguous) |
| Author | Scicluna B | Scicluna B (not ambiguous) |
| Author | Wang Y / Wang, Y / Wang Yan / Wang Ying … | Wang Y (ambiguous aggregate) |
| Author | Zhang Y / Zhang, Y / Zhang Yu / Zhang Ying … | Zhang Y (ambiguous aggregate) |

**Panel B. Top journals (after normalization)**

| Rank | Journal | Publications |
| --- | --- | --- |
| 1 | Frontiers in Immunology | 108 |
| 2 | Shock | 86 |
| 3 | Critical Care | 73 |
| 4 | Critical Care Medicine | 62 |
| 5 | International Journal of Molecular Sciences | 44 |
| 6 | PLOS One | 41 |
| 7 | Chinese Critical Care Medicine | 38 |
| 8 | Journal of Clinical Medicine | 33 |
| 9 | Scientific Reports | 32 |
| 10 | Frontiers in Medicine | 30 |

**Panel C. Top countries (full counting)**

| Rank | Country | Publications |
| --- | --- | --- |
| 1 | United States | 939 |
| 2 | China | 557 |
| 3 | United Kingdom | 262 |
| 4 | Germany | 249 |
| 5 | Netherlands | 191 |
| 6 | France | 173 |
| 7 | Canada | 147 |
| 8 | Italy | 141 |
| 9 | Spain | 96 |
| 10 | India | 93 |

**Panel D. Top institutions after normalization**

| Rank | Institution | Publications |
| --- | --- | --- |
| 1 | University of Pittsburgh | 76 |
| 2 | Jena University Hospital | 55 |
| 3 | University of Amsterdam / Amsterdam UMC | 54 |
| 4 | University of Athens | 44 |
| 5 | Radboud University Medical Center | 26 |
| 6 | Karolinska Institute | 20 |
| 7 | University Medical Center Utrecht | 16 |
| 8 | University of Oxford | 16 |
| 9 | University of Pennsylvania | 9 |
| 10 | Capital Medical University | 5 |

**Panel E. Top author-name strings (surname-initial; ambiguity flag)**

| Author-name string | Publications | Potential ambiguity |
| --- | --- | --- |
| Van Der Poll T | 106 | No |
| Wang Y | 47 | Yes |
| Scicluna B | 46 | No |
| Giamarellos-Bourboulis E | 43 | No |
| Wiersinga W | 42 | No |
| Cremer O | 40 | No |
| Schultz M | 38 | No |
| Wang J | 38 | Yes |
| Zhang Y | 37 | Yes |
| Van Vught L | 37 | No |
| Moldawer L | 36 | No |
| Sweeney T | 35 | No |
| Chen Y | 34 | Yes |
| Bauer M | 33 | No |
| Bonten M | 32 | No |

Rankings were recalculated after metadata normalization and after correcting Web of Science continuation-line author parsing. Institution rankings were calculated after representative variant-to-canonical harmonization. Wang Y, Wang J, Zhang Y, and Chen Y are flagged as potentially ambiguous author-name aggregates.

## **Supplementary Table 5. Annual topic-count matrix for the 2000-2025 subset.**

| Year | Topic 1 | Topic 2 | Topic 3 | Topic 4 | Topic 5 | Topic 6 | Topic 7 | Topic 8 | Total |
| --- | --- | --- | --- | --- | --- | --- | --- | --- | --- |
| 2000 | 14 | 0 | 0 | 2 | 3 | 0 | 1 | 2 | 22 |
| 2001 | 21 | 1 | 2 | 3 | 5 | 1 | 0 | 0 | 33 |
| 2002 | 14 | 1 | 0 | 0 | 3 | 3 | 0 | 1 | 22 |
| 2003 | 18 | 0 | 1 | 2 | 4 | 2 | 1 | 1 | 29 |
| 2004 | 7 | 2 | 3 | 3 | 4 | 1 | 0 | 3 | 23 |
| 2005 | 16 | 0 | 1 | 1 | 4 | 4 | 2 | 3 | 31 |
| 2006 | 13 | 0 | 1 | 3 | 1 | 5 | 3 | 2 | 28 |
| 2007 | 19 | 2 | 1 | 4 | 4 | 2 | 4 | 3 | 39 |
| 2008 | 23 | 0 | 1 | 7 | 5 | 6 | 6 | 1 | 49 |
| 2009 | 21 | 3 | 1 | 5 | 6 | 7 | 2 | 5 | 50 |
| 2010 | 22 | 2 | 0 | 7 | 9 | 7 | 2 | 8 | 57 |
| 2011 | 21 | 3 | 3 | 4 | 10 | 6 | 5 | 3 | 55 |
| 2012 | 22 | 1 | 2 | 6 | 9 | 6 | 1 | 3 | 50 |
| 2013 | 19 | 5 | 2 | 3 | 3 | 6 | 0 | 2 | 40 |
| 2014 | 29 | 4 | 2 | 9 | 7 | 12 | 2 | 0 | 65 |
| 2015 | 21 | 1 | 5 | 8 | 10 | 7 | 7 | 2 | 61 |
| 2016 | 19 | 11 | 8 | 19 | 9 | 18 | 16 | 4 | 104 |
| 2017 | 27 | 10 | 4 | 22 | 20 | 22 | 17 | 1 | 123 |
| 2018 | 34 | 13 | 5 | 32 | 17 | 17 | 12 | 1 | 131 |
| 2019 | 30 | 13 | 20 | 41 | 17 | 20 | 17 | 4 | 162 |
| 2020 | 35 | 28 | 17 | 39 | 27 | 19 | 17 | 5 | 187 |
| 2021 | 48 | 42 | 26 | 44 | 39 | 31 | 10 | 4 | 244 |
| 2022 | 42 | 33 | 24 | 48 | 36 | 29 | 17 | 3 | 232 |
| 2023 | 36 | 40 | 33 | 63 | 29 | 25 | 20 | 0 | 246 |
| 2024 | 43 | 41 | 42 | 65 | 27 | 36 | 11 | 1 | 266 |
| 2025 | 32 | 51 | 51 | 89 | 47 | 36 | 18 | 1 | 325 |

The 2000-2025 display subset contains 2,674 records. The final analyzed corpus contains 2,974 records; records outside this display window were retained in the overall corpus and topic-size calculations but were not included in this annual matrix. Each annual row total equals the sum of its eight topic counts.

## **Supplementary Table 6. LDA, preprocessing, topic-assignment, temporal-display, and network-visualization parameters.**

| Category | Parameter | Value |
| --- | --- | --- |
| Data source / corpus | Documents | 2,974 |
| Data source / corpus | Text fields | title + abstract + author keywords + index keywords |
| Data source / corpus | Vocabulary size | 6,083 |
| Data source / corpus | Document-term matrix | 2,974 x 6,083 |
| Preprocessing | Lowercase | yes |
| Preprocessing | URLs removed | yes |
| Preprocessing | Non-alphabetic symbols removed | yes |
| Preprocessing | Standard English stopwords removed | yes |
| Preprocessing | Query terms removed | sepsis, septic, shock |
| Preprocessing | Demographic MeSH/check-tag noise removed | yes |
| Preprocessing | Lemmatization | no |
| Preprocessing | Minimum token length | 3 |
| Preprocessing | no_below | 5 |
| Preprocessing | no_above | 0.5 |
| LDA model | Software | gensim 4.4.0; LdaModel class |
| LDA model | Final K | 8 |
| LDA model | random_state | 42 |
| LDA model | passes | 20 |
| LDA model | iterations | 200 |
| LDA model | alpha | auto |
| LDA model | eta | auto |
| Model selection | K range | 4-12 |
| Model selection | Repeated seeds | 42, 7, 2026 |
| Model selection | Diagnostics | c_v coherence, log-perplexity, seed stability, minimum topic size |
| Model selection | K=8 selection | balanced compromise (c_v=0.5184; stability=0.3775; min_topic_size=83.0; best c_v at K=12; best stability at K=4) |
| Topic assignment | Assignment method | dominant topic = argmax of document-topic posterior probability |
| Topic assignment | Full posterior saved | yes (topic_1..topic_8 probabilities per document) |
| Temporal displays | Full analyzed corpus | 2,974 |
| Temporal displays | Records outside 2000-2025 display window | 300 |
| Temporal displays | 2000-2025 display subset | 2,674 |
| Temporal displays | Main trend display | 2010-2025 |
| Temporal displays | Supplementary annual matrix | 2000-2025 |
| Network visualization | General: Software / package | Python 3.10 + networkx (graph construction); matplotlib (rendering) |
| Network visualization | General: Counting method | full counting (each occurrence counted once per record) |
| Network visualization | Country network: Country counting rule | each country counted once per publication (per-record dedup) |
| Network visualization | Country network: Edge definition | co-occurrence of two countries within the same publication |
| Network visualization | Country network: Edge weight | number of co-authored publications shared by the country pair |
| Network visualization | Country network: Layout | force-directed (spring) layout |
| Network visualization | Keyword network: Keyword source | author keywords + index keywords / keywords plus (combined) |
| Network visualization | Keyword network: Keyword normalization | lowercase, whitespace/punctuation harmonization, singular/plural consolidation |
| Network visualization | Keyword network: Minimum keyword frequency | >=10 occurrences for display |
| Network visualization | Keyword network: Edge definition | co-occurrence within the same publication |
| Network visualization | Keyword network: Edge-weight normalization | association strength (normalized co-occurrence) |
| Network visualization | Keyword overlay: Node color meaning | average publication year of documents containing the keyword |
| Network visualization | Keyword overlay: Layout | force-directed (spring) layout |

## **Supplementary Table 7. Preprocessing sensitivity analyses (K = 8).**

| Pipeline | Vocabulary size | c_v coherence (K=8) | Log-perplexity (K=8) | Top-20 term overlap vs primary | Dominant-assignment agreement vs primary | Macro-themes recognizable |
| --- | --- | --- | --- | --- | --- | --- |
| Primary | 6083 | 0.5156 | -7.4038 | 1.0 | 1.0 | reference |
| Sensitivity A (stemming) | 4168 | 0.4968 | -6.9415 | 0.102 | 0.444 | partly |
| Sensitivity B (retain shock) | 6084 | 0.4934 | -7.4134 | 0.319 | 0.407 | partly |
| Sensitivity C (retain all query terms) | 6085 | 0.5179 | -7.3727 | 0.372 | 0.441 | partly |
| Sensitivity D (keep demographic terms) | 6099 | 0.5011 | -7.4015 | 0.44 | 0.559 | partly |

Each alternative pipeline was compared with the primary pipeline at K = 8 using a single model per pipeline (random_state = 42). Macro-level themes remained interpretable, but topic composition was preprocessing-dependent: top-20 term overlap ranged 0.10-0.44 and dominant-assignment agreement ranged 0.41-0.56 after best topic matching. LDA topics should be interpreted as exploratory thematic structures rather than fixed categories. Morphological normalization in Sensitivity A used Porter/Snowball stemming.

## **Supplementary Table 8. Adjudication of same-title records after DOI-based deduplication.**

| group_id | normalized_title | n_before_adjudication | n_after_adjudication | n_distinct_norm_doi | merge_or_retain_decision | rationale |
| --- | --- | --- | --- | --- | --- | --- |
| G001 | 20 years on is it time to redefine the systemic inflammatory response to cardiothoracic surgery | 2 | 1 | 0 | merge (single work) | A2:same first-author + year (within identical normalized title) |
| G002 | a new definition of sepsis sepsis 3 aims advantages and controversies | 2 | 2 | 0 | retain all (distinct works) | no merge rule satisfied (distinct first-author or year); differing first author |
| G003 | a novel acute phase marker in cattle lipopolysaccharide binding protein lbp | 2 | 1 | 1 | merge (single work) | A2:same first-author + year (within identical normalized title) |
| G004 | acute on chronic liver failure | 2 | 2 | 2 | retain all (distinct works) | no merge rule satisfied (distinct first-author or year); differing publication year; differing first author |
| G005 | adrenal insufficiency in sepsis | 2 | 2 | 1 | retain all (distinct works) | no merge rule satisfied (distinct first-author or year); differing publication year; differing first author |
| G006 | beneficial effect of enhanced macrophage function in the trauma patient | 2 | 1 | 0 | merge (single work) | A2:same first-author + year (within identical normalized title) |
| G007 | cationic antimicrobial peptides and their multifunctional role in the immune system | 3 | 1 | 1 | merge (single work) | A2:same first-author + year (within identical normalized title) |
| G008 | cd16 monocytes in patients with cancer spontaneous elevation and pharmacologic induction by recombinant human | 2 | 1 | 1 | merge (single work) | A2:same first-author + year (within identical normalized title) |
| G009 | combined biomarker panel of presepsin he4 and oxygenation index for sepsis diagnosis and prognosis in intensiv | 2 | 1 | 1 | merge (single work) | A2:same first-author + year (within identical normalized title) |
| G010 | development and prospective implementation of a large language model based system for early sepsis prediction | 2 | 1 | 2 | merge (single work) | A2:same first-author + year (within identical normalized title) |
| G011 | development and validation of a cellular host response test as an early diagnostic for sepsis | 2 | 1 | 1 | merge (single work) | A2:same first-author + year (within identical normalized title) |
| G012 | early inhibition of activated fibrinolysis predicts microbial infection shock and mortality in febrile medical | 2 | 1 | 1 | merge (single work) | A2:same first-author + year (within identical normalized title) |
| G013 | elevated red cell distribution width as a prognostic marker in severe sepsis a prospective observational study | 2 | 1 | 2 | merge (single work) | A1:loose-DOI match |
| G014 | endotoxin in the pathogenesis of sepsis | 2 | 1 | 1 | merge (single work) | A2:same first-author + year (within identical normalized title) |
| G015 | endotoxin stimulates platelet derived growth factor production from cultured human pulmonary endothelial cells | 2 | 1 | 1 | merge (single work) | A2:same first-author + year (within identical normalized title) |
| G016 | endotoxins and other sepsis triggers | 2 | 1 | 1 | merge (single work) | A2:same first-author + year (within identical normalized title) |
| G017 | epidemiology of adult population sepsis in india a single center 5 year experience | 2 | 1 | 2 | merge (single work) | A1:loose-DOI match |
| G018 | exploring the diagnostic and prognostic significance of alkaline phosphatase on neutrophil surface membrane in | 2 | 1 | 0 | merge (single work) | A2:same first-author + year (within identical normalized title) |
| G019 | gram negative bacterial sepsis and sepsis syndrome | 2 | 1 | 1 | merge (single work) | A2:same first-author + year (within identical normalized title) |
| G020 | host innate immune responses to microbial pathogens | 3 | 1 | 1 | merge (single work) | A2:same first-author + year (within identical normalized title) |
| G021 | host response to sars cov 2 insight from transcriptomic studies | 2 | 1 | 0 | merge (single work) | A2:same first-author + year (within identical normalized title) |
| G022 | host responses in mediating sepsis and adult respiratory distress syndrome | 2 | 1 | 0 | merge (single work) | A2:same first-author + year (within identical normalized title) |
| G023 | il 8 in septic shock endotoxemia and after il 1 administration | 2 | 2 | 0 | retain all (distinct works) | no merge rule satisfied (distinct first-author or year); differing first author |
| G024 | immunosuppression in the surgical patient | 2 | 1 | 0 | merge (single work) | A2:same first-author + year (within identical normalized title) |
| G025 | induction of circulating group ii phospholipase a2 expression in adults with malaria | 2 | 1 | 1 | merge (single work) | A2:same first-author + year (within identical normalized title) |
| G026 | infective endocarditis update experience from a heart hospital | 2 | 1 | 0 | merge (single work) | A2:same first-author + year (within identical normalized title) |
| G027 | interleukin 1 and tumor necrosis factor and their naturally occurring antagonists during hemodialysis | 2 | 1 | 0 | merge (single work) | A2:same first-author + year (within identical normalized title) |
| G028 | lemierre s syndrome in a patient with severe lupus nephritis | 3 | 1 | 1 | merge (single work) | A2:same first-author + year (within identical normalized title) |
| G029 | malnutrition injury and the host immune response nutrient substitution | 2 | 1 | 0 | merge (single work) | A2:same first-author + year (within identical normalized title) |
| G030 | mesenchymal stromal cell therapies potential and pitfalls for ards | 3 | 2 | 0 | partial merge | A2:same first-author + year (within identical normalized title) |
| G031 | multiple systems organ failure failure of host defense homeostasis | 2 | 1 | 1 | merge (single work) | A2:same first-author + year (within identical normalized title) |
| G032 | nampt associated gene signature in the prediction of severe sepsis | 2 | 1 | 0 | merge (single work) | A2:same first-author + year (within identical normalized title) |
| G033 | novel insights into the regulatory role of n6 methyladenosine methylation modified autophagy in sepsis | 2 | 1 | 1 | merge (single work) | A2:same first-author + year (within identical normalized title) |
| G034 | optimization of genomic classifiers for clinical deployment evaluation of bayesian optimization to select pred | 2 | 1 | 0 | merge (single work) | A2:same first-author + year (within identical normalized title) |
| G035 | organ dysfunction in sepsis an ominous trajectory from infection to death | 3 | 1 | 0 | merge (single work) | A2:same first-author + year (within identical normalized title) |
| G036 | pathogenic effects of endotoxin | 2 | 1 | 0 | merge (single work) | A2:same first-author + year (within identical normalized title) |
| G037 | pathophysiology of sepsis | 4 | 4 | 4 | retain all (distinct works) | no merge rule satisfied (distinct first-author or year); generic title -> retained as distinct works by differing author/year; differing publication year; differing first author |
| G038 | pathophysiology of sepsis and septic shock | 2 | 1 | 0 | merge (single work) | A2:same first-author + year (within identical normalized title) |
| G039 | physiologic support of the septic patient | 2 | 1 | 1 | merge (single work) | A2:same first-author + year (within identical normalized title) |
| G040 | predicting hospital readmission among patients with sepsis using clinical and wearable data | 2 | 1 | 2 | merge (single work) | A2:same first-author + year (within identical normalized title) |
| G041 | procalcitonin how a hormone became a marker and mediator of sepsis | 3 | 1 | 1 | merge (single work) | A2:same first-author + year (within identical normalized title) |
| G042 | procalcitonin s role in the sepsis cascade is procalcitonin a sepsis marker or mediator | 3 | 1 | 0 | merge (single work) | A2:same first-author + year (within identical normalized title) |
| G043 | protective effects of activated protein c in sepsis | 2 | 1 | 1 | merge (single work) | A2:same first-author + year (within identical normalized title) |
| G044 | recent insights into the pathogenesis of bacterial sepsis | 3 | 1 | 0 | merge (single work) | A2:same first-author + year (within identical normalized title) |
| G045 | relationship between sepsis induced immunosuppression and multi drugs resistant bacteria | 2 | 1 | 2 | merge (single work) | A1:loose-DOI match |
| G046 | role of biomarkers in patients with dyspnea | 3 | 1 | 0 | merge (single work) | A2:same first-author + year (within identical normalized title) |
| G047 | role of cholinergic anti inflammatory pathway in regulating host response and its interventional strategy for | 2 | 1 | 1 | merge (single work) | A2:same first-author + year (within identical normalized title) |
| G048 | role of sepsis modulated circulating micrornas | 2 | 1 | 0 | merge (single work) | A2:same first-author + year (within identical normalized title) |
| G049 | sepsis | 7 | 7 | 7 | retain all (distinct works) | no merge rule satisfied (distinct first-author or year); generic title -> retained as distinct works by differing author/year; differing publication year; differing first author |
| G050 | sepsis and host response | 5 | 5 | 0 | retain all (distinct works) | no merge rule satisfied (distinct first-author or year); differing publication year; differing first author |
| G051 | sepsis and identification of reliable biomarkers for postoperative period prognosis | 3 | 2 | 0 | partial merge | A2:same first-author + year (within identical normalized title) |
| G052 | sepsis and multiple organ failure | 4 | 2 | 3 | partial merge | A2:same first-author + year (within identical normalized title); generic title -> retained as distinct works by differing author/year |
| G053 | sepsis and septic shock | 6 | 6 | 6 | retain all (distinct works) | no merge rule satisfied (distinct first-author or year); generic title -> retained as distinct works by differing author/year; differing publication year; differing first author |
| G054 | sepsis and septic shock sepsis und septischer schock | 5 | 2 | 0 | partial merge | A2:same first-author + year (within identical normalized title) |
| G055 | sepsis and the liver | 2 | 2 | 2 | retain all (distinct works) | no merge rule satisfied (distinct first-author or year); differing publication year; differing first author |
| G056 | sepsis diagnosis and management | 3 | 2 | 0 | partial merge | A2:same first-author + year (within identical normalized title) |
| G057 | sepsis endotypes identified by host gene expression across global cohorts | 2 | 2 | 1 | retain all (distinct works) | no merge rule satisfied (distinct first-author or year); differing publication year |
| G058 | sepsis from bench to bedside | 2 | 1 | 1 | merge (single work) | A2:same first-author + year (within identical normalized title) |
| G059 | sepsis new insights new definition sepsis nieuwe inzichten nieuwe definitie | 2 | 2 | 0 | retain all (distinct works) | no merge rule satisfied (distinct first-author or year); differing first author |
| G060 | septic shock | 4 | 4 | 4 | retain all (distinct works) | no merge rule satisfied (distinct first-author or year); generic title -> retained as distinct works by differing author/year; differing publication year; differing first author |
| G061 | studies on the inflammatory coagulant axis in the baboon response to e coli regulatory roles of proteins c s c | 2 | 1 | 0 | merge (single work) | A2:same first-author + year (within identical normalized title) |
| G062 | subpopulations of neutrophils with increased oxidative product formation in blood of patients with infection | 2 | 1 | 0 | merge (single work) | A2:same first-author + year (within identical normalized title) |
| G063 | the cardiovascular effects of sepsis | 2 | 1 | 1 | merge (single work) | A2:same first-author + year (within identical normalized title) |
| G064 | the development of anti inflammatory drugs for infectious diseases | 3 | 1 | 0 | merge (single work) | A2:same first-author + year (within identical normalized title) |
| G065 | the evolution of the understanding of sepsis infection and the host response a brief history | 2 | 2 | 2 | retain all (distinct works) | no merge rule satisfied (distinct first-author or year); differing publication year |
| G066 | the gut as a potential trigger of exercise induced inflammatory responses | 2 | 1 | 2 | merge (single work) | A2:same first-author + year (within identical normalized title) |
| G067 | the hypothalamic pituitary adrenal axis in sepsis | 3 | 2 | 1 | partial merge | A2:same first-author + year (within identical normalized title) |
| G068 | the inflammatory caspases key players in the host response to pathogenic invasion and sepsis | 2 | 2 | 1 | retain all (distinct works) | no merge rule satisfied (distinct first-author or year); differing publication year; differing first author |
| G069 | the inflammatory coagulant axis in the host response to gram negative sepsis regulatory roles of proteins and | 2 | 1 | 0 | merge (single work) | A2:same first-author + year (within identical normalized title) |
| G070 | the lipemia of sepsis triglyceride rich lipoproteins as agents of innate immunity | 2 | 1 | 1 | merge (single work) | A2:same first-author + year (within identical normalized title) |
| G071 | the role of immunostimulatory nucleic acids in septic shock | 3 | 2 | 0 | partial merge | A2:same first-author + year (within identical normalized title) |
| G072 | the role of platelets in sepsis | 2 | 2 | 2 | retain all (distinct works) | no merge rule satisfied (distinct first-author or year); differing publication year; differing first author |
| G073 | tolerization of inflammatory gene expression | 2 | 1 | 2 | merge (single work) | A2:same first-author + year (within identical normalized title) |
| G074 | treml4 a potential target for immunotherapy of sepsis | 2 | 1 | 0 | merge (single work) | A2:same first-author + year (within identical normalized title) |
| G075 | two hit hypothesis and multiple organ dysfunction syndrome | 3 | 1 | 1 | merge (single work) | A2:same first-author + year (within identical normalized title) |
| G076 | ubiquitous expression of the calcitonin i gene in multiple tissues in response to sepsis | 2 | 1 | 2 | merge (single work) | A2:same first-author + year (within identical normalized title) |
| G077 | urosepsis | 2 | 2 | 1 | retain all (distinct works) | no merge rule satisfied (distinct first-author or year); differing publication year; differing first author |
| G078 | urosepsis pathogenesis and treatment | 2 | 2 | 2 | retain all (distinct works) | no merge rule satisfied (distinct first-author or year); differing publication year |

Decisions: 54 groups merged to a single work, 7 partial merges, 17 groups retained as multiple distinct works. No retained multi-record group had a single first author and year; none met the predefined criteria for merging.

## **Supplementary Figure 1. Annual evolution of the eight LDA-defined thematic domains, 2000-2025.**


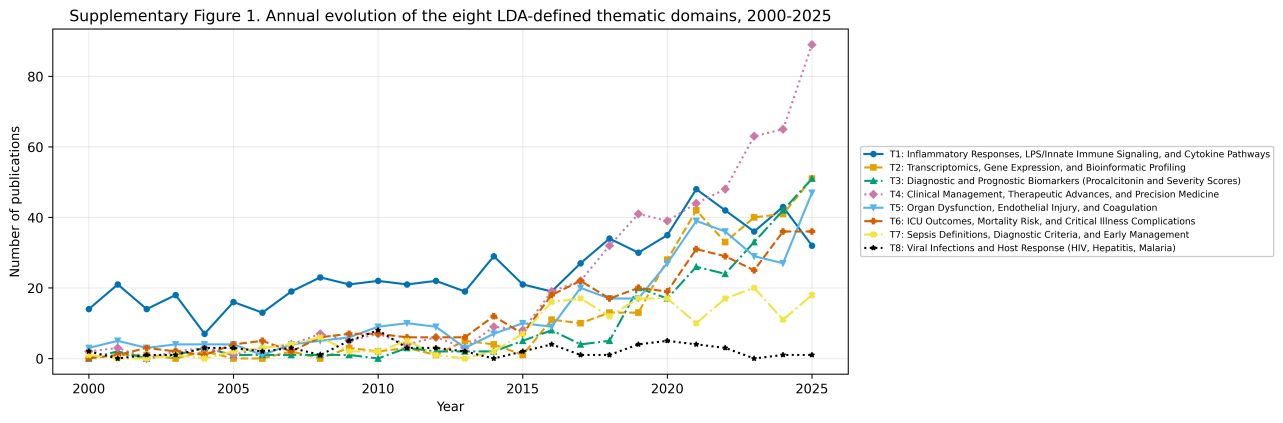


Supplementary Figure 1

Annual topic-specific publication counts derived from the dominant topic assignment on the final analyzed corpus.

## **Supplementary Figure 2. Publication output of leading countries.**


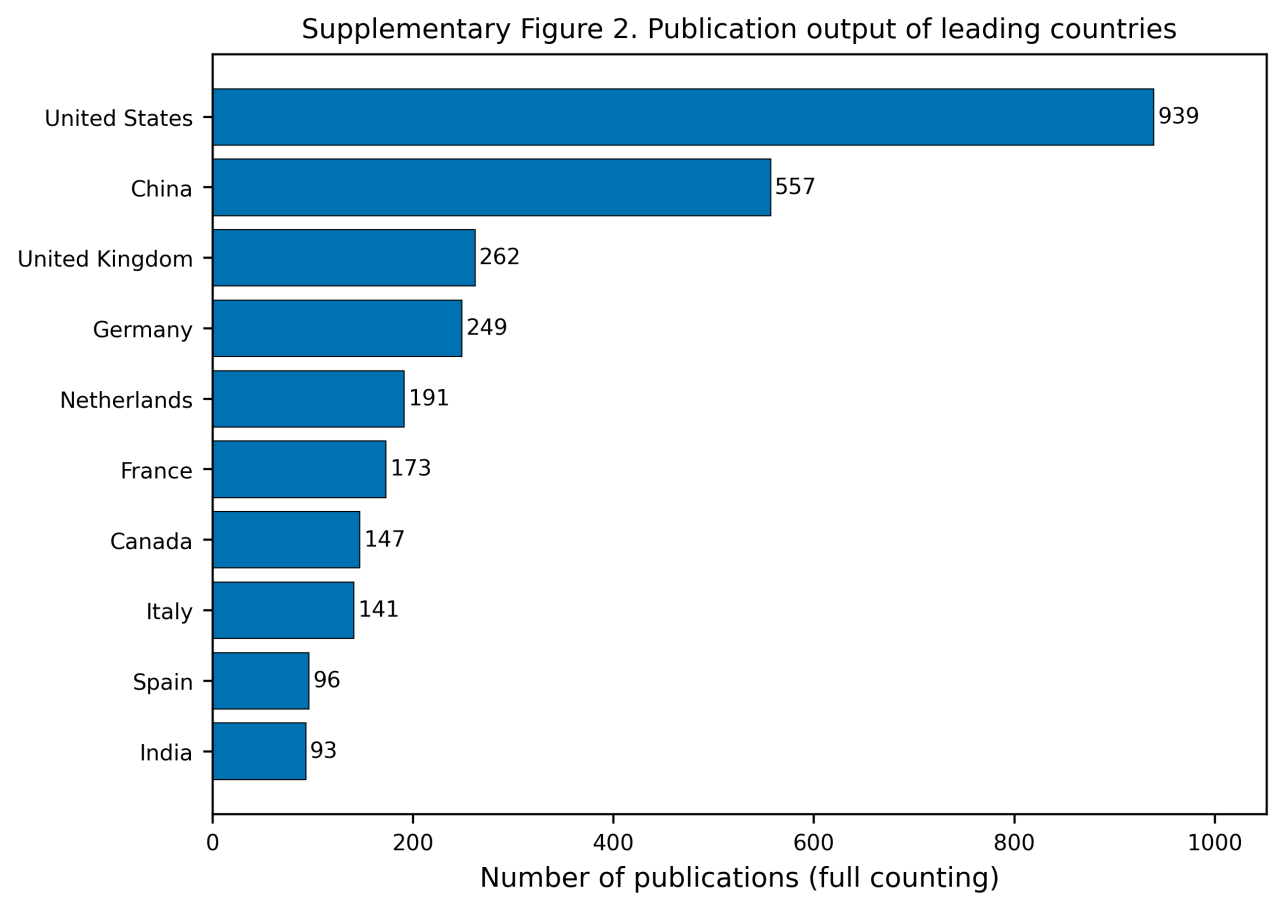


Supplementary Figure 2

Country-level publication output (full counting), complementing the country collaboration network in main-text Figure 5.

## **Supplementary Figure 3. Three-field plot among countries, journals, and LDA-defined thematic domains.**


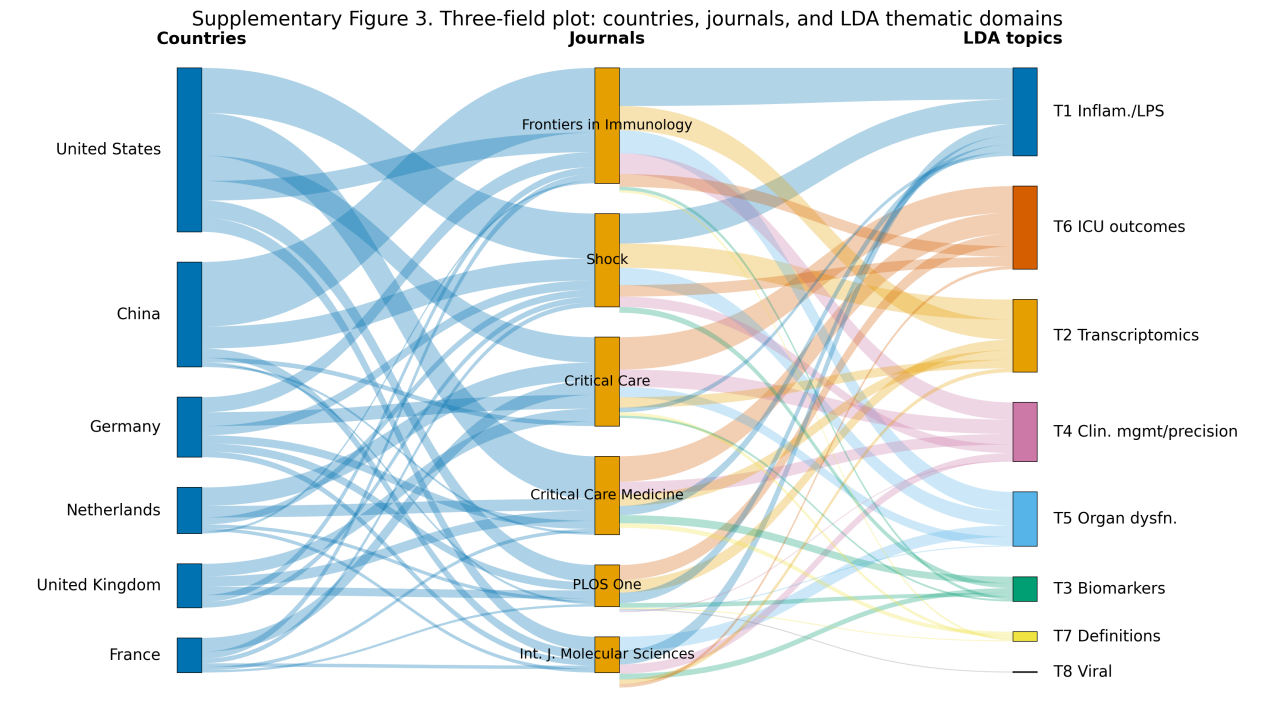


Supplementary Figure 3

Three-field plot linking the leading countries, journals, and the eight LDA topics; ribbon width is proportional to the number of shared publications.
